# Transcriptional and post-translational changes in the brain of mice deficient in cholesterol removal mediated by cytochrome P450 46A1 (CYP46A1)

**DOI:** 10.1371/journal.pone.0187168

**Published:** 2017-10-26

**Authors:** Natalia Mast, Joseph B. Lin, Kyle W. Anderson, Ingemar Bjorkhem, Irina A. Pikuleva

**Affiliations:** 1 Department of Ophthalmology and Visual Sciences, Case Western Reserve University, Cleveland, Ohio, United States of America; 2 Biomolecular Measurement Division, National Institute of Standards and Technology, Gaithersburg, Maryland, United States of America; 3 Institute for Bioscience and Biotechnology Research, Rockville, Maryland, United States of America; 4 Department of Laboratory Medicine, Division of Clinical Chemistry, Karolinska Institute, Huddinge, Sweden; Universite Clermont Auvergne, FRANCE

## Abstract

Cytochrome P450 46A1 (CYP46A1) converts cholesterol to 24-hydroxycholesterol and thereby controls the major pathways of cholesterol removal from the brain. *Cyp46a1*^*-/-*^ mice have a reduction in the rate of cholesterol biosynthesis in the brain and significant impairments to memory and learning. To gain insights into the mechanisms underlying *Cyp46a1*^*-/-*^ phenotype, we used *Cyp46a1*^*-/-*^ mice and quantified their brain sterol levels and the expression of the genes pertinent to cholesterol homeostasis. We also compared the *Cyp46a1*^*-/-*^ and wild type brains for protein phosphorylation and ubiquitination. The data obtained enable the following inferences. First, there seems to be a compensatory upregulation in the *Cyp46a1*^*-/-*^ brain of the pathways of cholesterol storage and CYP46A1-independent removal. Second, transcriptional regulation of the brain cholesterol biosynthesis *via* sterol regulatory element binding transcription factors is not significantly activated in the *Cyp46a1*^*-/-*^ brain to explain a compensatory decrease in cholesterol biosynthesis. Third, some of the liver X receptor target genes (*Abca1*) are paradoxically upregulated in the *Cyp46a1*^*-/-*^ brain, possibly due to a reduced activation of the small GTPases RAB8, CDC42, and RAC as a result of a reduced phosphorylation of RAB3IP and PAK1. Fourth, the phosphorylation of many other proteins (a total of 146) is altered in the *Cyp46a1*^*-/-*^ brain, including microtubule associated and neurofilament proteins (the MAP and NEF families) along with proteins related to synaptic vesicles and synaptic neurotransmission (e.g., SLCs, SHANKs, and BSN). Fifth, the extent of protein ubiquitination is increased in the *Cyp46a1*^*-/-*^ brain, and the affected proteins pertain to ubiquitination (UBE2N), cognition (STX1B and ATP1A2), cytoskeleton function (TUBA1A and YWHAZ), and energy production (ATP1A2 and ALDOA). The present study demonstrates the diverse potential effects of CYP46A1 deficiency on brain functions and identifies important proteins that could be affected by this deficiency.

## Introduction

**Fig 1:**
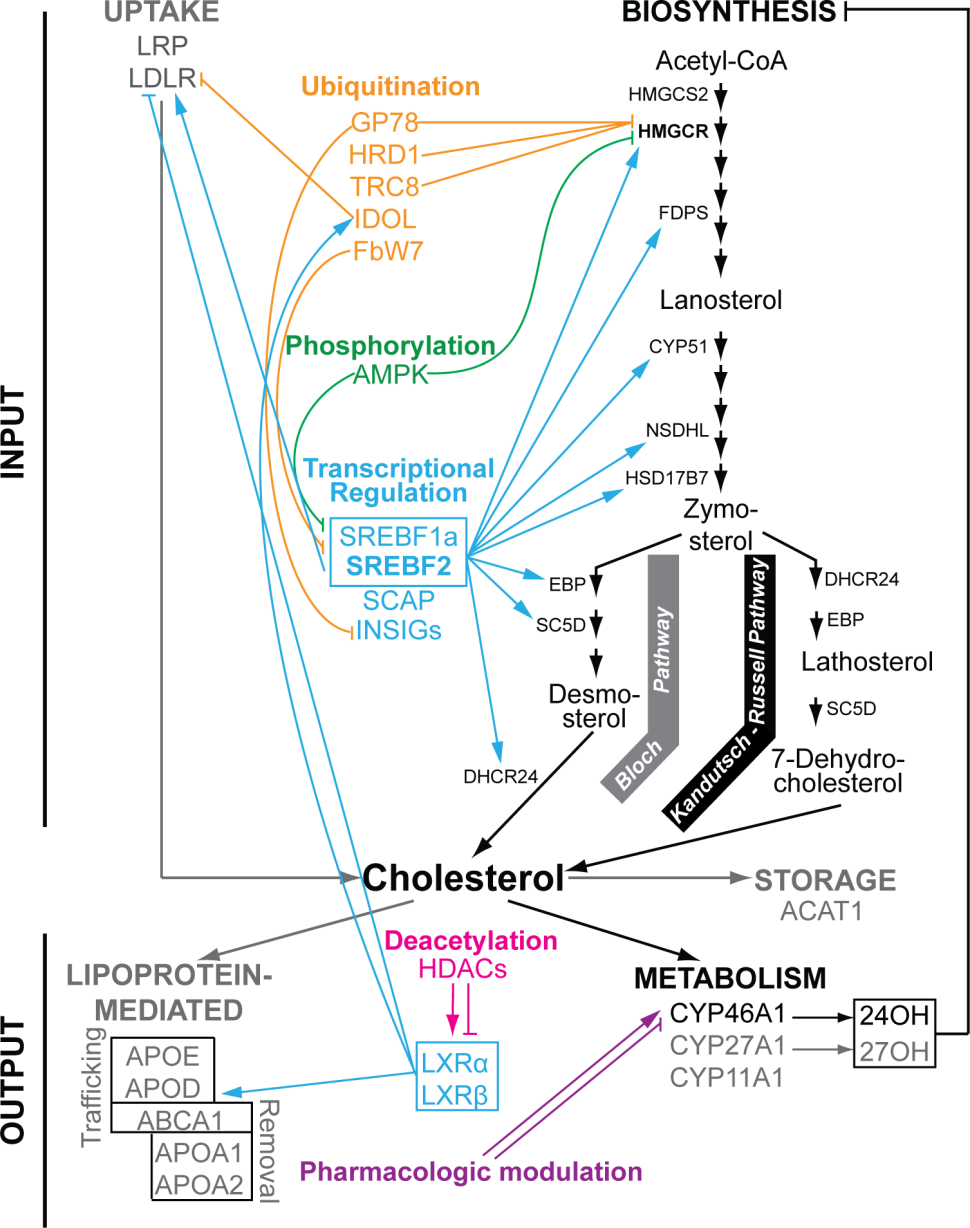
Cholesterol homeostasis. Simplified representation of the pathways of brain cholesterol input, output, and storage along with potential mechanisms regulating these pathways. The pathways of major and minor quantitative significance are in black bold and gray bold, respectively. Not all the proteins in these pathways and regulatory mechanisms are indicated, mostly those that were studied or discussed in the present work. Different regulatory mechanisms are indicated in different colors. Arrows indicate activation, blunt-ends denote repression or inhibition. Protein symbols are deciphered in S1 Text. 24OH, 24-hydroxycholesterol, the product of CYP46A1; 27OH, 27-hydroxycholesterol, the product of CYP27A1.

The brain is a cholesterol-rich organ and has a tightly regulated cholesterol homeostasis, which balances the pathways of cholesterol input and output [1]. The major pathway of brain cholesterol input is local biosynthesis (Fig 1), a source of essentially all brain cholesterol [1]. Cholesterol biosynthesis involves many steps and requires at least 19 different enzymes to carry out these reactions; HMGCR (S1 Text) is the rate-limiting enzyme in this pathway [2]. Brain cholesterol output is also realized mainly *via* one pathway, namely cholesterol 24-hydroxylation catalyzed by cytochrome P450 46A1 (CYP46A1) [3, 4]. Unlike cholesterol, 24-hydroxycholesterol can cross the blood-brain barrier and rapidly diffuse into the systemic circulation for subsequent biotransformations in the liver [5]. The brain pathway of cholesterol removal independent of CYP46A1 exists as well [6] but is not fully understood and believed to utilize the ABCA1 transporter and apolipoprotein particles containing APOA1 [7]. Cholesterol biosynthesis in neurons is thought to be insufficient for the cell maintenance and effective synapse formation; hence additional cholesterol is required and believed to be supplied to neurons by astrocytes *via* lipoprotein-mediated transport [8]. Thus, there is cholesterol trafficking and exchange in the brain between different cell types. These processes are mediated by ABCA1 (which effluxes cholesterol outside a cell), apolipoproteins APOE, APOJ, and APOD (that transport the effluxed cholesterol to a different cell type) as well as cellular receptors LDLR and LRP (that uptake cholesterol-containing lipoprotein particles) [7]. In addition, the brain has the capacity to store a small amount of cholesterol excess intracellularly, in lipid droplets, after esterification by ACAT1 [9, 10].

Very little is currently known about the regulation of cholesterol homeostasis in the brain, and it is unclear whether different cell types in the brain have the same mechanisms of cellular cholesterol maintenance. Evidence has accumulated indicating that some of the brain cell types probably regulate their cholesterol biosynthesis at the level of transcription controlled by the isoforms of SREBF [10]. SREBF2 activates the expression of many genes in cholesterol biosynthesis, whereas SREBF1a enhances both cholesterol and fatty acid biosynthesis [11]. Both transcription factors are synthesized as ~120 kDa precursors that are anchored to the endoplasmic reticulum (ER) through the interactions with the escort protein SCAP and an ER retention protein INSIG [12]. When cholesterol levels are low, precursor SREBFs are released from the ER and transported to the Golgi by SCAP, where they are cleaved to release active SREBF (~68 kDa), which can then enter the nucleus and activate the transcription of the target genes [13]. Cells outside the brain are known to regulate cholesterol biosynthesis post-translationally. Phosphorylation of SREBF2, SREBF1c, and HMGCR by a protein kinase AMPK inhibits transcriptional activity of SREBFs and enzyme activity of HMGCR [14, 15]. Also, a decrease in HMGCR activity and protein levels of INSIGs, SREBFs, as well as LDLR could be achieved by ubiquitin-mediated degradation involving ubiquitin ligases GP78, HRD1, TRC8, FBW7, and IDOL [16]. It is thus possible, yet needs to be proven, that in addition to transcriptional regulation, cholesterol biosynthesis in the brain could be regulated post-translationally utilizing the same mechanisms that are operative outside the brain.

Studies in mice suggest that similar to other organs, both transcriptional and post-translational regulation is operative in the brain to control the pathways of cholesterol output. Cholesterol trafficking and transport from the brain seem to be regulated, at least in part, at the level of transcription by the LXR isoforms α and β [6, 17, 18], which are activated by the cholesterol precursor desmosterol and side-chain oxidized oxysterols produced from cholesterol by the cytochrome P450 enzymes CYP46A1, CYP27A1, and CYP11A1 [19–21]. Side-chain oxysterols upregulate the LXR target genes (e.g., *Abca1*, *Apoe*, *Apod*, and *Idol*) in a gene-, cell- and tissue-specific manner [22, 23], and in the brain, do not seem to be the general regulators of LXRs [24, 25]; perhaps they could activate LXRs under specific conditions and/or in certain cell types. 24- and 27-Hydroxycholesterols are proposed to be physiological suppressors of the brain cholesterol biosynthesis [25], the contention supported by increased cholesterol synthesis in the brain of mice with a reduced brain level of 24-hydroxycholesterol as a consequence of increased metabolism [25] or increased leakage across the blood brain barrier [26]. In the liver and cancer cells, transcriptional activity of LXRs was shown to be modulated by the histone deacetylases HDACs, which deacetylate LXRs. Subsequent effect on the expression of the LXR target genes is, however, specific to the HDAC and LXR isoforms [27, 28]. The transcriptional control of CYP46A1 initiating the major pathway of cholesterol removal from the brain appears to be weak. *Cyp46a1* transcription was found to be insensitive to major regulatory axes, except oxidative stress [29], and controlled by the Sp transcription factors at the level of basal expression [30]. The Sp-mediated *Cyp46a1* basal expression seems to be affected by recruitment/detachment of HDACs 1 and 2 to the *Cyp46a1* promoter region and concomitant histone deacetylation/acetylation [31–34]. So far, the only mechanism that was shown to modulate CYP46A1 activity *in vivo* was the pharmacologic activation or inhibition of the enzyme by marketed drugs [35–37]. Some endogenous compounds, like a major neurotransmitter glutamate, could activate CYP46A1 post-translationally as well [38, 39].

The present work was initiated by the finding that the brain of *Cyp46a1*^*-/-*^ mice has the same steady state levels of cholesterol but a reduced rate of cholesterol biosynthesis (by ~40–50%) to compensate for a lack of the major pathway of cholesterol output [40]. Remarkably, *Cyp46a1*^*-/-*^ mice also have severe deficiencies in spatial, associative, and motor learning attributed to impaired long-term potentiation (LTP), a result of reduced protein prenylation with non-steroidal cholesterol precursors [41, 42]. We decided to gain insight into the processes in the *Cyp46a1*^*-/-*^ brain underlying a reduction in the cholesterol biosynthesis rate and assessed the major transcriptional and post-translational mechanisms of cholesterol homeostatic regulation. With several exceptions, changes in the gene transcription were moderate in the *Cyp46a1*^*-/-*^ brain, yet there were significant and meaningful effects on protein phosphorylation and ubiquitination.

## Methods

### Animals

*Cyp46a1*^*-/-*^ mice were obtained from Dr. David Russell (UT Southwestern) [43] and were on the mixed C57BL/6J;129S6/SvEv background. Mice were 3–10 months old, kept on a 12-hour light-dark cycle, and provided standard rodent chow and water *ad libitum*. All animal experiments were approved by Case Western Reserve University’s Institutional Animal Care and Use Committee and conformed to recommendations made by the American Veterinary Association Panel on Euthanasia.

### Sterol quantification by gas chromatography-mass spectrometry

Brain hemispheres from 3–5 month old mice (n = 3) were homogenized individually and processed as described [44], except sterols were derivatized with 100 μl bis-(trimethylsilyl) trifluoroacetamide/trimethylchlorosilane. Internal standards (the deuterated analogs of the measured sterols) were added to 1 mg of brain protein (1 g of wet brain contains approximately 115 mg of protein) prior to extraction of lipids by Folch. The following internal standards were used: [26,26,26,27,27,27-^2^H_6_]lanosterol, [2,2,4,4-^2^H_4_]lathosterol, [26,26,26,27,27,27-^2^H_6_]desmosterol, [2,2,3,4,4-^2^H_5_]zymosterol, [25,26,26,26,27,27,27-^2^H_7_]cholesterol, [25,26,26,26,27,27,27-^2^H_7_]24(*R/S*)-hydroxycholesterol, [26,26,26,27,27-^2^H_5_]27-hydroxycholesterol, [17α,21,21,21-^2^H_4_]pregnenolone, and [25,26,26,26,27,27,27-^2^H_7_]7-ketocholesterol. 7-Dehydrocholesterol was quantified using [2,2,4,4-^2^H_4_]lathosterol as the internal standard. One nmol of internal standard was used for all sterols, except 500 nmol was used for [25,26,26,26,27,27,27-^2^H_7_]cholesterol. Deuterated analogs of lathosterol, 27-hydroxycholesterol, and pregnenolone were obtained from Medical Isotopes, Inc. (Pelham NH) while deuterated analogs of all other sterols were obtained from Avanti Polar Lipids, Inc. (Alabaster AL). Cholesterol was measured as total (a sum of free and esterified) and free cholesterol, while other sterols were measured in the free form only. Derivatized sterol samples were analyzed by an Agilent 5973 Network Mass Selective Detector equipped with an Agilent 6890 Gas Chromatograph system and a ZB-5MS capillary column (60 m × 0.25 mm × 0.25 mm; Zebron, Charlotte, NC). The mass spectrometer was operated in selected ion monitoring mode using electron impact ionization, and gas chromatograph conditions for monitoring of sterols were as described [44]. For quantifications of total and free cholesterol, 0.2 μl of the trimethylsilylated sample was injected into the GC-MS in split mode (1:20). For quantifications of all other free sterols, 1 μl was injected in splitless mode. The following ions were monitored: 393 (lanosterol), 399 ([^2^H_6_]lanosterol), 458 (lathosterol), 462 ([^2^H_4_]lathosterol), 366 (desmosterol), 372 ([^2^H_6_]desmosterol), 456 (zymosterol), 461 ([^2^H_5_]zymosterol), 368 (cholesterol), 375 ([^2^H_7_]cholesterol), 145 (24-hydroxycholesterol), 152 ([^2^H_7_]24(*R/S*)-hydroxycholesterol), 417 (27-hydroxycholesterol), 422 ([^2^H_5_]27-hydroxycholesterol), 388 (pregnenolone), 392 ([^2^H_4_]pregnenolone), and 351 (7-dehydrocholesterol). Abundances for each ion fragment were calculated by the maximum peak height. Quantifications were performed using calibration curves generated using a fixed concentration of internal standard and varying concentrations of unlabeled sterol.

### RNA isolation and cDNA synthesis

Total RNA was isolated from 3–4 month old mice. For PCR array, 3 brain hemispheres from 3 different mice were processed as a pooled sample, while for quantitative real-time PCR (qRT-PCR), brain hemispheres from 3–6 mice were processed individually. Pooled or individual tissues were homogenized in 10 volumes (w/v) of TRIzol (Life Technologies). Total RNA was extracted following the manufacturer’s procedure (Life Technologies). One μg of the isolated RNA was used for cDNA synthesis using SuperScript III Reverse Transcriptase (Invitrogen) to be then used for gene profiling by PCR array and qRT-PCR.

### PCR arrays

cDNA was used for gene profiling by the Mouse Lipoprotein Signaling & Cholesterol Metabolism PCR Array (PAMM-080Z, Qiagen) and a Mastercycler RealPlex^2^ PCR machine (Eppendorf). Ct numbers for genes of interest were normalized to the expression of 5 housekeeping genes (*Actb*, *B2m*, *Gapdh*, *Gusb*, and *Hsp90ab1*) and converted into fold changes relative to the corresponding genes in the wild type strain using the manufacturer’s web-based software (RT^2^ Profiler PCR Array Data Analysis version 3.5).

### Quantitative real-time PCR (qRT-PCR)

Sequences of primers for gene quantifications by qRT-PCR were taken from qPrimerDepot, a primer database for qRT-PCR [45], and provided in S1 Table. PCR reactions were performed using samples from individual animals (n = 3–6) in duplicate using a LightCycler 96 PCR system (Roche); data were normalized to expression of *Actb*. Primer quality for individual genes was assessed by confirming that the melting curves had a single peak.

### Protein quantification by Western blot

SREBF2 was measured in brain and liver lysates prepared from tissues of 3–4 month old female mice. Brain (one hemisphere) or liver (1/2 of the liver) was shredded by drawing the tissue through an 18 G needle for 25 strokes, and placed in 10 volumes (w/v) of 50 mM Tris-HCl buffer (pH 7.4) containing 1 mM EDTA, 0.5 mM EGTA, 0.5% sodium deoxycholate (w/v), 0.1% SDS (w/v), 1% NP-40 (v/v), 150 mM NaCl, 50 mM NaF, 1 mM PMSF, and a cocktail of protease inhibitors. After solubilization on ice for 30 min, lysates were centrifuged at 14,000 x *g* at 4°C for 15 min, and the supernatant was isolated. Samples were added to a mixture of 2X Laemmli Sample Buffer (Bio-Rad) and 2-mercaptoethanol (95:5, v/v) and heated for 10 min at 90°C. Ten microgram of protein of brain lysates or 20 μg of protein of liver lysates were loaded per lane of a 10% Mini-PROTEAN TGX Precast Gel (Bio-Rad). SDS-PAGE was run at 100 V for 1 hour then 130 V for 20 min at room temperature in 1X Tris/Glycine/SDS running buffer (Bio-Rad). After SDS-PAGE, proteins were transferred onto an Odyssey nitrocellulose membrane (LI-COR) at 100 V for 1 hour and blocked for 1 hour using 5% nonfat dry milk (w/v, Bio-Rad) in 1X phosphate-buffered saline (PBS). The membrane was then incubated at 4°C overnight with a primary antibody cocktail containing 0.1% Tween-20 (v/v), rabbit polyclonal antibody against SREBF2 (Novus Biological NBP2-20481, dilution 1:500), and mouse monoclonal antibody against α-tubulin (Abcam ab7291, dilution 1:20,000). The membrane was then washed 6 times with 1X PBS containing 0.1% Tween-20 (v/v), incubated for 1 hour at room temperature with a secondary antibody cocktail, which was followed by another set of 6 washes. The secondary antibody cocktail contained 0.1% Tween-20 (v/v), goat anti-mouse secondary antibody (IRDye 800CW, LI-COR, dilution 1:5,000), and goat anti-rabbit secondary antibody (IRDye 680RD, LI-COR, dilution: 1:5,000). Fluorescent secondary antibodies were detected using an Odyssey Fc Imaging System (LI-COR), and SREBF2 bands were quantified using Image Studio Software (LI-COR) and normalized to the intensity of α-tubulin.

HMGCR was measured in brain microsomes prepared from tissues of 6-month old male mice. One brain hemisphere was homogenized manually using a Teflon pestle in 10 volumes (w/v) of 50 mM Tris-HCl buffer (pH 7.4) containing 250 mM sucrose, 5 mM MgCl_2_, 1 mM dithiothreitol, 1 mM phenylmethylsulfonyl fluoride, 100 μg/ml butyl hydroxytoluene, and a cocktail of protease inhibitors. Homogenates were centrifuged at 9,000 x *g* for 20 min at 4°C to pellet unbroken cells, nuclei, cell debris, and mitochondria. The supernatant was isolated and centrifuged at 106,000 x *g* for 60 min at 4°C to pellet the microsomes, which were then resuspended in 100 μl 50 mM potassium phosphate buffer (pH 7.2) containing 1 mM EDTA. Immunoblotting was performed following a similar procedure to that used for SREBF2, except 20 μg protein of brain microsomes were loaded per lane, casein blocking buffer was used (LI-COR), and antibody cocktails contained 0.25% Tween-20 (v/v). HMGCR was detected using a rabbit polyclonal antibody (United States Biological H6201-01C, dilution 1:250), and normalized to the intensity of β-actin detected by a mouse monoclonal antibody (Santa Cruz Biotechnology Inc., dilution 1:500).

### Protein quantification by liquid chromatography-mass spectrometry

Each sample was prepared from a brain hemisphere of a 3-month old female mouse. Tissue was added to 2 ml of 50 mM ammonium bicarbonate, pH 7.8, supplemented with 1:200 (v/w) protease inhibitor cocktail (P1860, Sigma-Aldrich) and sonicated three times at 10% intensity for 10 seconds (Sonicator 3000, Misonix Inc.) on ice. Total protein was measured using Pierce BCA protein assay kit (Thermo Fisher Scientific), and 10 mg of each homogenate was sampled for analysis. Samples were centrifuged at 106,000 *g* for 1 hour at 4°C, and the resulting supernatant and pellet fractions were processed separately. For measurement of HDACs, supernatant fractions were supplemented with 25 mM ammonium bicarbonate/0.5% SDS (v/w)/20 mM dithiothreitol/5 pmol of ^15^N-labeled QconCATs for HDACs [46]. For measurement of ABCA1, pellet fractions were reconstituted with 25 mM ammonium bicarbonate/2% SDS (v/w)/20 mM dithiothreitol/3 pmol of ^15^N-labeled ABCA1. After incubation for 1 hour at room temperature for reduction of cysteine residues, all samples were alkylated by treatment with 55 mM iodoacetamide for 1 hour. Sequential protein precipitation with a chloroform/methanol/water mixture (12.5:50:37.5, v/v/v) and then with four volumes of methanol was performed [47] to separate proteins from salts, lipids, and processing reagents. Precipitated protein samples were reconstituted in 25 mM ammonium bicarbonate/0.2% sodium cholate (v/w) and digested with 1:10 trypsin (Type IX-S, Sigma-Aldrich) to protein ratio (w/w) overnight at 37°C. Digested sample was quenched with 0.5% trifluoroacetic acid and centrifuged at 106,000 *g* for 20 min at 4°C. Supernatant was dried using Eppendorf AG Vacufuge, and dried peptides were stored at -20°C. For analysis by liquid chromatography-mass spectrometry, dried peptides were reconstituted in an acetonitrile/water mixture (3:97, v/v, containing 0.1% formic acid (v/v), and multiple reaction monitoring (MRM) measurements were conducted on an Agilent 6490 iFunnel triple quadrupole, as previously described [48]. MRM transitions were predicted using Pinpoint software (Thermo Fisher Scientific) for both unlabeled and ^15^N-labeled peptides. Precursors with +2 charge were selected for analysis. Transitions were screened in a digest of ^15^N-labeled standard to obtain the 3 or 4 most intense transitions, which were further used for quantification (Table 1). Relative signal ratios of transitions for quantification were similar in both the ^15^N-labeled standard digest and when standards were added into tissue homogenates, indicating no obvious interference from biological matrices on the quantification using selected transitions. Quantification was performed by calculating the ratio of peak areas for unlabeled biological peptides to labeled standard peptides using MassHunter software (Agilent) multiplying by the ratio of known picomoles of standard to milligrams of total protein. MRM data from 3–4 transitions per peptide were combined to determine amount of protein, presented as mean ± SD. For HDAC4, transitions from two peptides were all combined to obtain protein abundance, while other HDACs and ABCA1 used only one peptide.

**Table 1:** Peptides and transitions for quantification of ABCA1 and HDACs by mass spectrometry.

Protein	Peptide		Precursor (m/z)	Product ions (m/z)			
ABCA1	ILYTPDTPATR	L	624.3	545.3 (y_5_)	757.4 (y_7_)	858.4 (y_8_)	
		H	631.3	553.3 (y_5_)	767.4 (y_7_)	869.4 (y_8_)	
HDAC1,2	YGEYFPGTGDLR	L	687.8	715.4 (y_7_)	862.4 (y_8_)	1025.5 (y_9_)	
		H	695.3	725.3 (y_7_)	873.4 (y_8_)	1037.5 (y_9_)	
HDAC3	YTGASLQGATQLNNK	L	783.4	845.4 (y_8_)	973.5 (y_9_)	1173.6 (y_11_)	
		H	793.4	857.4 (y_8_)	987.5 (y_9_)	1189.6 (y_11_)	
HDAC4	ESAVASTEVK	L	510.8	563.3 (y_5_)	634.3 (y_6_)	733.4 (y_7_)	
		H	516.2	569.3 (y_5_)	641.3 (y_6_)	741.4 (y_7_)	
	DQPVELLNPAR	L	626.3	683.4 (y_6_)	812.5 (y_7_)	1008.6 (y_9_)	
		H	634.3	693.4 (y_6_)	823.4 (y_7_)	1021.5 (y_9_)	
HDAC5	LSTQQEAER	L	510.8	632.3 (y_5_)	760.4 (y_6_)	861.4 (y_7_)	
		H	531.3	641.3 (y_5_)	771.3 (y_6_)	873.4 (y_7_)	
HDAC6	EQLIQEGLLDR	L	657.4	702.4 (y_6_)	830.4 (y_7_)	943.5 (y_8_)	1056.6 (y_9_)
		H	665.3	711.4 (y_6_)	841.4 (y_7_)	955.5 (y_8_)	1069.6 (y_9_)

Transitions are listed for both unlabeled, light (L) and ^15^N-labeled, heavy (H) peptides. Precursor ions are +2 charge and product ions are +1 charge with y-ion included for reference.

### Brain phosphoproteomics

Each sample was prepared from a brain hemisphere of a 8–10 month old female mouse lysed in 0.5 ml of 20 mM HEPES, pH 8.0, containing 9 M urea and 1X Halt phosphatase inhibitor cocktail (Thermo Scientific). Brain lysates were reduced and alkylated with 10 mM dithiothreitol and 30 mM iodoacetamide, respectively, and the concentration of urea in these lysates was decreased to <2 M by dilution with 20 mM HEPES. Next, trypsin was added at a protein to trypsin ratio of 1:50–1:100 (w/w), and the protein digests were spiked with three synthetic phosphopeptides (angiotensin II, cholecystokinin, and calcitonin, PS-180-1, Protea Biosciences). Sample enrichment with phosphoserine- and phosphothreonine-containing peptides was carried out using Thermo Scientific Pierce TiO_2_ Phosphopeptide Enrichment and Clean-up Kit (PI88301) according to the manufacturer’s instructions. Enrichment efficiency was assessed by monitoring the three synthetic phosphopeptides. Enriched peptide samples were subjected to clean-up by C18 cartridges (360 mg, WAT05190, Waters Corporation) and were subsequently analyzed by a Finnigan LTQ-Obitrap Elite hybrid mass spectrometer connected to a Dionex Acclaim Pepmap C18 reversed-phase capillary chromatography column (15 cm x 75 μm, 2 μm particle size, 100 Å pores). Five microliters of the extracts were injected, and peptides were eluted at a flow rate of 0.25 μl/min using a gradient of solvent B in solvent A: 5% B for 5 min, 5% to 40% B in 115 min, 40% to 80% B in 12 min, 80% B for 5 min, 80% to 5% B in 1 min, and 5% B for 14 min. Solvent A was water containing 0.1% formic acid (v/v), and solvent B was acetonitrile containing 5% water (v/v) and 0.1% formic acid (v/v). The eluted peptides were analyzed by mass spectrometry with the following parameters: microelectrospray ion source 1.9 kV. The digest was analyzed using the data dependent multitask capability of the instrument acquiring full scan mass spectra to determine peptide molecular weights and product ion spectra to determine amino acid sequence in successive instrument scans. The data were analyzed by using all CID spectra collected in the experiment to search the mouse reference sequence databases with the program Sequest that is bundled in Proteome Discoverer 1.4 (Thermo Scientific) considering oxidized methionine along with serine, threonine, and tyrosine phosphorylation as a dynamic modification. Carbamidomethylation at cysteine residues was considered as a static modification. The resulting search results were filtered based on an Sequest XCorr scores > 1.5 (+1), 2.0 (+2), 2.25 (+3), and 2.5 (+4). The quantitation of these phosphorylated peptides was performed by a label free method that involved aligning the LC chromatograms and comparing the extracted ion chromatograms for each identified peptide by using the program SIEVE (Thermo Scientific). Data were normalized to the total ion chromatogram. All differentially expressed phosphopeptides were manually validated using a second normalization step to the synthetic phosphopeptides added as internal standards. Differentially-abundant phosphorylated proteins in the *Cyp46a1*^*-/-*^ brain were analyzed by the GeneAnalytics (LifeMap Sciences) software.

### Brain protein ubiquitination

Brain hemispheres from 8–10 month old female mice were processed following a procedure similar to that used for preparation of brain samples for the phospoproteome analysis, except 5 mg protein was digested with trypsin, and samples were enriched with K-GlyGly using PTMScan K-ε-GG kit (5562, Cell Signaling) according to the manufacturer’s instructions. Enriched peptides were subjected to clean-up by C18 cartridges (360 mg, WAT05190, Waters Corporation) and were subsequently analyzed by a Finnigan LTQ-Obitrap Elite hybrid mass spectrometer connected to a Dionex Acclaim Pepmap C18 reversed-phase capillary chromatography column (15 cm x 75 μm, 2 μm particle size, 100 Å pores). Five microliters of the extracts were injected and peptides were eluted at a flow rate of 0.25 μl/min by a gradient of acetonitrile in water containing 0.1% formic acid (v/v). The gradient was: 2% to 70% acetonitrile (v/v) in 150 min. The eluted peptides were analyzed by mass spectrometry with the following parameters: microelectrospray ion source 1.9 kV. The digest was analyzed using the data dependent multitask capability of the instrument acquiring full scan mass spectra to determine peptide molecular weights and product ion spectra to determine amino acid sequence in successive instrument scans. The data were analyzed by using all CID spectra collected in the experiment to search the human reference sequence databases with the program Sequest considering GlyGly modification at Lysine residues. The resulting search results were filtered based on Sequest XCorr scores > 1.5 (+1), 2.0 (+2), 2.25 (+3), and 2.5 (+4). The data from these samples was searched against the full mouse database considering K + GlyGly as a dynamic modification. The quantitation of these ubiquitinated peptides was performed by a label free method that involves aligning the LC chromatograms and comparing the extracted ion chromatograms for each identified peptide and was performed using the program SIEVE. Data were normalized to the total ion chromatogram.

### Statistical analysis

Data represent mean ± SD. Statistical significance for differences in sterol measurements was assessed by first performing a two-way ANOVA. If differences were found to be significant based on genotype, gender, or the interaction of genotype and gender, then individual pairwise comparisons were assessed for significance using Bonferroni correction. Statistical significances for differences in gene expression and protein quantifications were assessed using a two-tailed, unpaired Student’s t-test. Statistical significance was defined as *, *P*≤0.05; **, *P*≤0.01; ***, *P*≤0.001; ****, P≤0.0001; and *****, P≤0.00001.

## Results

### Brain sterol quantifications

**Fig 2:**
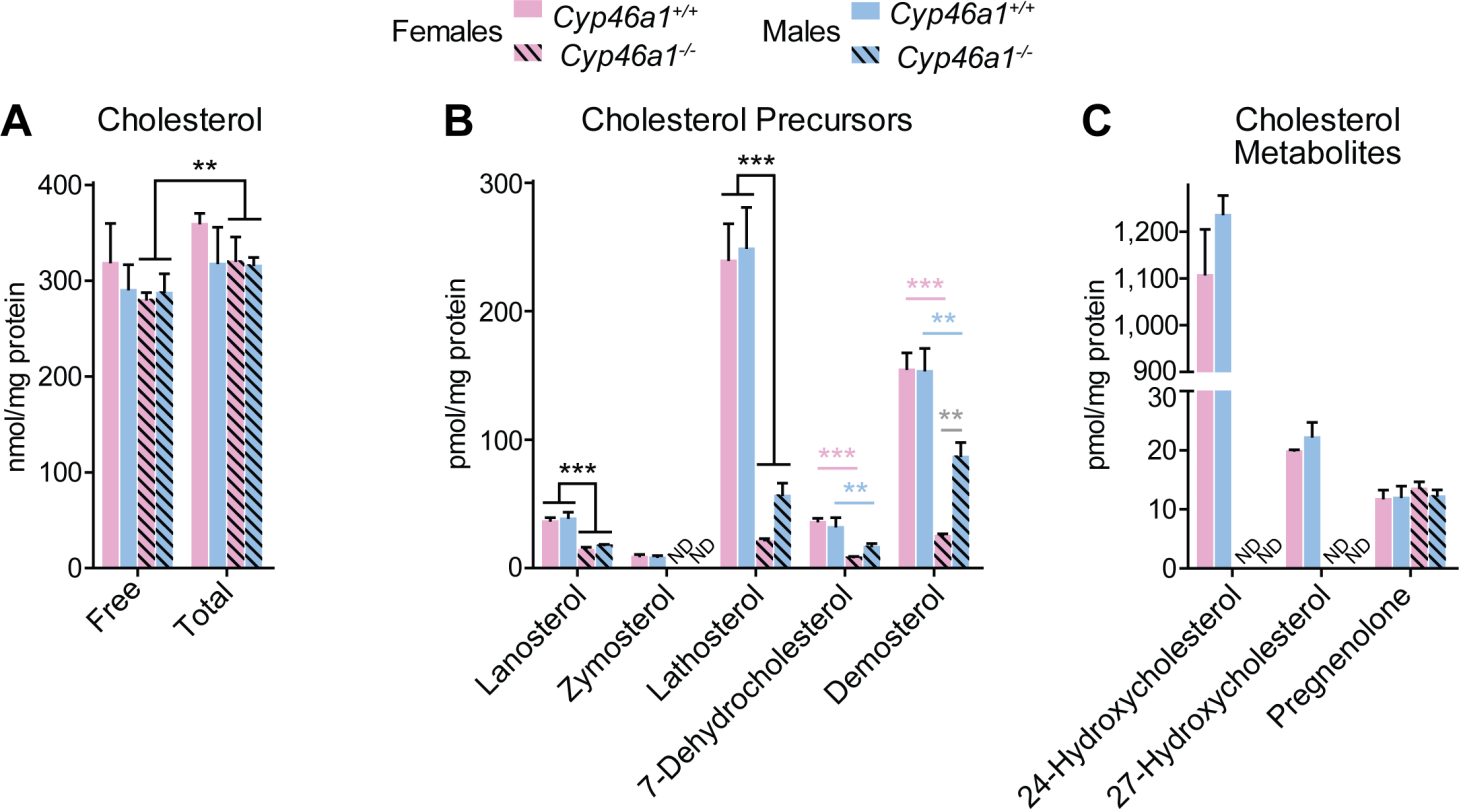
Sterol quantifications in the brain of *Cyp46a1*^*-/-*^ mice and wild type controls. Data for cholesterol, lathosterol, desmosterol, 24-hydroxycholesterol, and 27-hydroxycholesterol were taken from [49]. The data represent the mean ± SD of three measurements in individual (n = 3) 3–5 month old mice. Statistical significance was assessed two-way ANOVA followed by pairwise comparisons made using the Bonferroni correction. Significant comparisons of free sterols are denoted by the following color code: pink asterisks, significant changes between *Cyp46a1*^*-/-*^ females versus wild type females; blue asterisks, significant changes between *Cyp46a1*^*-/-*^ males versus wild type males; gray asterisks, significant changes between female and male mice of the same strain; and black asterisks, significant changes between the genotypes when data were collapsed across genders. *, P≤0.05; **, P≤0.01; ***, P≤0.001; and ND, not detectable (the limit of detection was 1 pmol/mg protein).

Previously, we characterized some of the brain sterols in *Cyp46a1*^*-/-*^ mice [49] and found that total brain cholesterol (i.e., a sum of free and esterified cholesterol) was unchanged in these animals as compared to wild type controls, yet the amount of esterified cholesterol was increased (Fig 2A). The levels of lathosterol and desmosterol, reflecting cholesterol biosynthesis in neurons (the Kandutsch-Russell pathway) and astrocytes (the Bloch pathway), respectively [7], were significantly decreased in the *Cyp46a1*^*-/-*^ brain, whereas 27-hydroxycholesterol was undetectable (Fig 2B and 2C) [49]. In the present work, three additional cholesterol precursors (lanosterol, zymosterol, and 7-dehydrocholesterol, Fig 2B) and one more cholesterol metabolite (pregnenolone) were quantified (Fig 2C). Similar to lathosterol and desmosterol, lanosterol and zymosterol, the upstream precursors of lathosterol and desmosterol in the pathway of cholesterol biosynthesis, were decreased in the *Cyp46a1*^*-/-*^ brain as is 7-dehydrocholesterol, a downstream sterol in this biosynthetic pathway. Thus, all the major sterol precursors in the cholesterol biosynthesis pathway were decreased in the *Cyp46a1*^*-/-*^ brain, consistent with a previously found decrease in the rate of cholesterol biosynthesis [40]. Female *Cyp46a1*^*-/-*^ mice showed the more pronounced decreases in the levels of many of cholesterol precursors than male animals, justifying the pregnenolone quantifications, which is the precursor and rate-limiting sterol in the production of all steroid hormones [50]. The pregnenolone amounts were similar and unchanged in *Cyp46a1*^*-/-*^ female and male mice, suggesting that their gender variability in the levels of brain cholesterol precursors is not due to a higher cholesterol utilization for the production of steroid hormones. Sterol quantifications in the brain provided the rationale for using either both *Cyp46a1*^*-/-*^ genders in subsequent experiments or mostly female mice, which had higher decreases in the brain cholesterol precursor levels.

### Gene expression as assessed by PCR array

**Fig 3:**
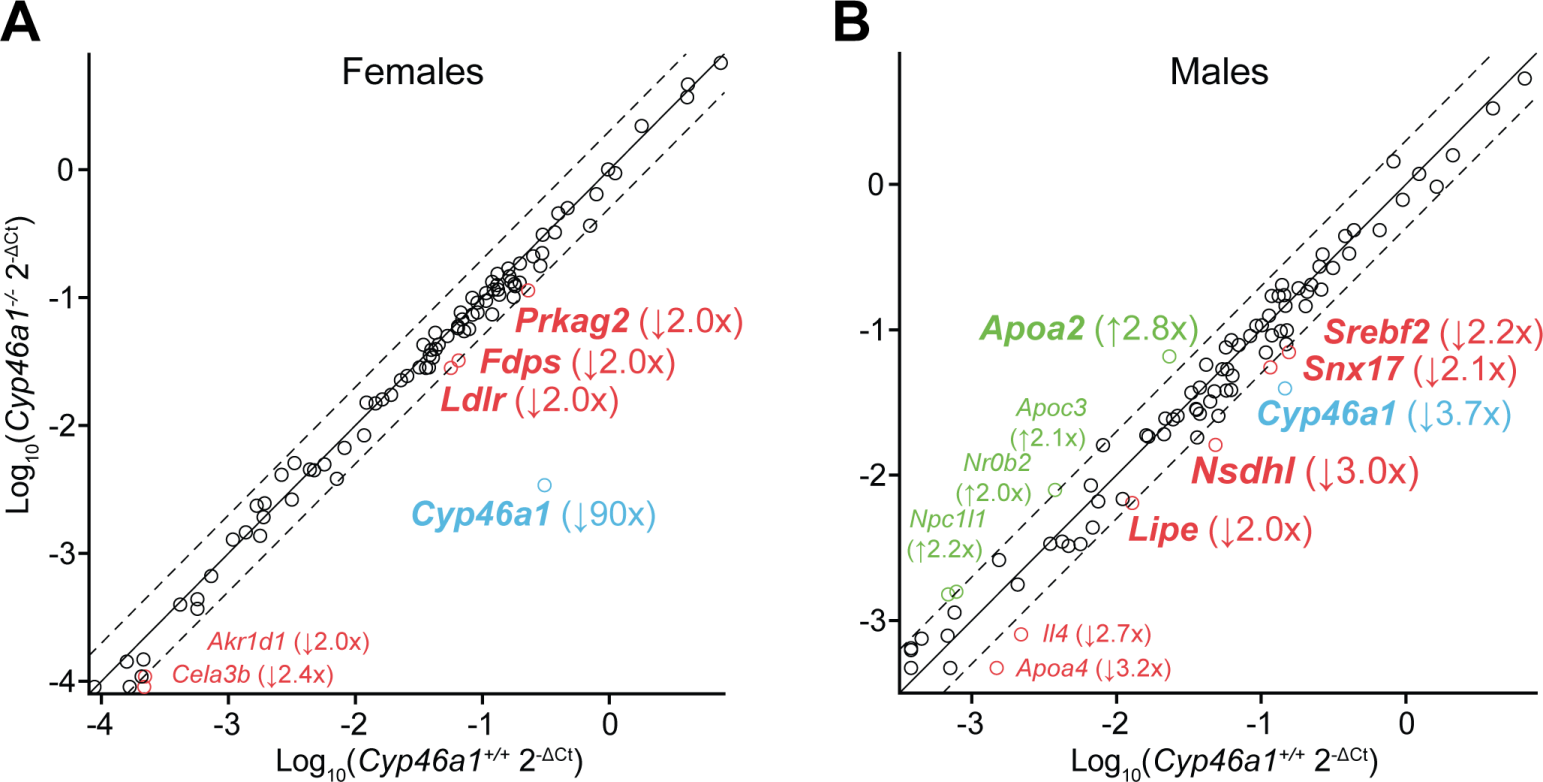
Profiling of cholesterol-related genes in the *Cyp46a1*^*-/-*^ brain by PCR array. A) Female 3–4 month old mice. B) Male 3–4 month old mice. For both female and male mice, a pooled sample of 3 brain hemispheres from different mice was used. Each circle indicates an individual gene in the PCR array. The up-regulated and down-regulated genes are in green and red, respectively (cutoff, 2.0, dashed line), and those with Ct numbers ≤ 30 are in bold. Genes in blue are those which were downregulated in both genders. The number in parenthesis indicates a fold change as compared to the wild type brain. Gene abbreviations are deciphered in S1 Text.

A total of 84 cholesterol-related genes were profiled by PCR array to gain insight into whether transcriptional regulation underlies wholly or in part a compensatory decrease in the *Cyp46a1*^*-/-*^ brain of cholesterol biosynthesis. All 84 genes in the array were detected. Of them, 6 genes in the brain of female *Cyp46a1*^*-/-*^ mice (*Prkag2*, *Fdps*, *Ldlr*, *Cyp46a1*, *Akr1d1*, and *Cela3b)* and 11 genes in the brain of male *Cyp46a1*^*-/-*^ mice (*Apoa2*, *Apoc3*, *Nr0b2*, *Npc1/1*, *Srebf2*, *Snx17*, *Cyp46a1*, *Nsdhl*, *Lipe*, *Il4*, and *Apoa4)* showed more than a 2-fold change (an arbitrary cut off limit) in the expression level as compared to the expression of the genes in the brain of wild type animals (Fig 3). Gene Ct numbers higher than 30 indicate very low, if any, protein expression. Accordingly, only the genes with Ct numbers ≤ 30 were then considered, 4 in female *Cyp46a1*^*-/-*^ mice (*Prkag2*, *Fdps*, *Ldlr*, and *Cyp46a1*), and 6 in male *Cyp46a1*^*-/-*^ mice (*Srebf2*, *Snx17*, *Cyp46a1*, *Nsdhl*, *Lipe*, and *Apoa2*). Of them, only one gene, *Cyp46a1*, was similarly downregulated in both genders, consistent with these animals’ genotype; all other genes with altered expression did not overlap in female and male *Cyp46a1*^*-/-*^ mice. In female *Cyp46a1*^*-/-*^ mice, all the affected genes were downregulated, including *Prkag2*, a non-catalytic subunit of AMPK related to post-translational regulation of cholesterol homeostasis as well as *Fdps* and *Ldlr*, the cholesterol input genes and targets of transcriptional regulation by SREBF [51]. In male *Cyp46a1*^*-/-*^ mice, there was both down- and upregulation of the affected genes. *Nsdhl*, the target of transcriptional regulation by SREBF2, was moderately downregulated as was *Srebf2* controlling cholesterol biosynthesis [51]. *Snx17* (sorting nexin 17) and *Apoa2* (apolipoprotein AII), related to intra- and extra-cellular cholesterol trafficking, respectively, were down- and up-regulated. Finally, *Lipe*, which de-esterifies either cholesterol esters in steroidogenic tissues or triglycerides in adipose tissue [52] was downregulated as well.

### Gene expression as assessed by qRT-PCR

**Fig 4:**
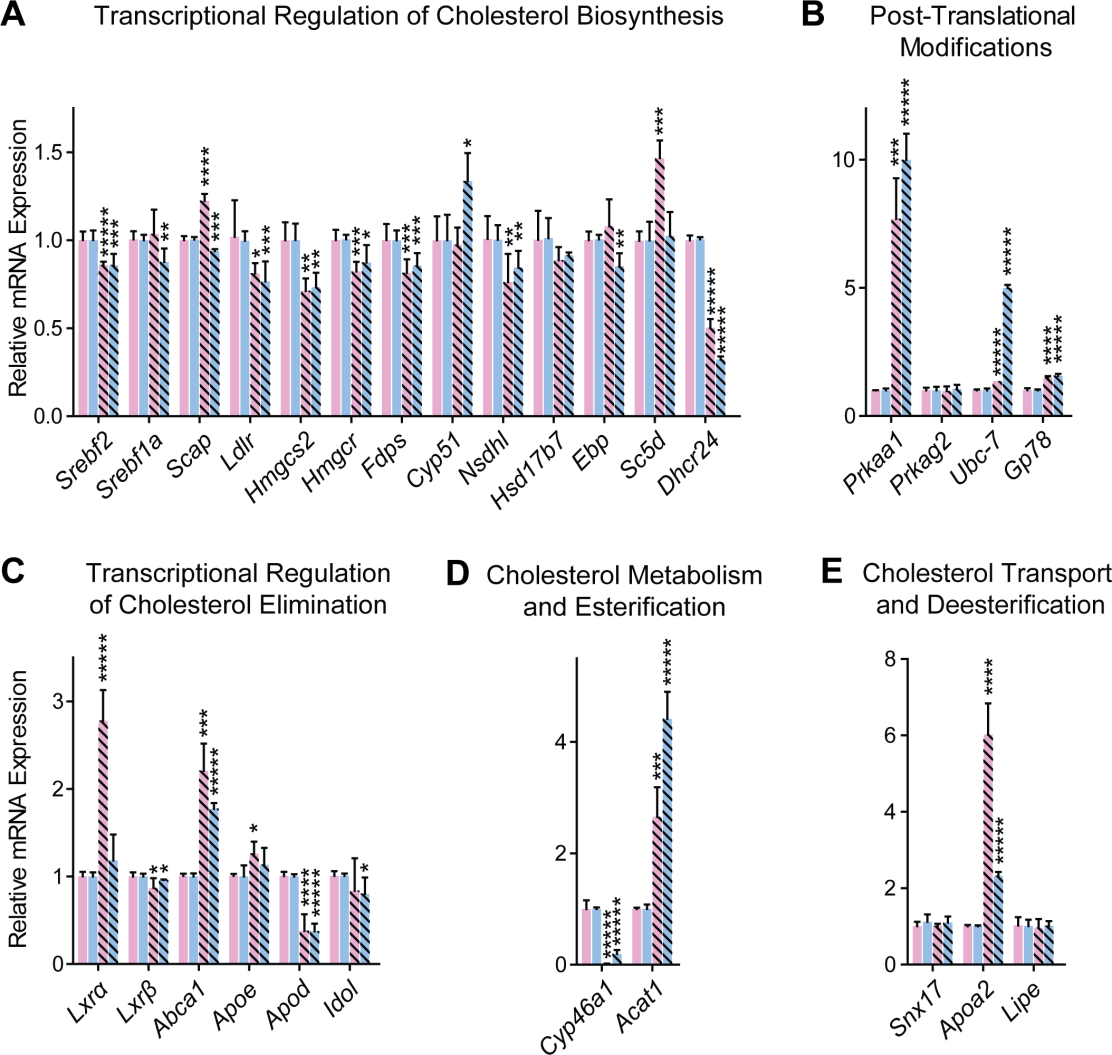
Evaluation of gene expression in the *Cyp46a1*^*-/-*^ brain by qRT-PCR. Genes are grouped based on function of the encoded proteins in cholesterol homeostasis. Each bar represents the mean ± SD of the PCR reactions performed individually on 3 to 6 animals in duplicate. The color code is the same as in Fig 2. Black asterisks indicate statistical significance between *Cyp46a1*^*-/-*^ and wild type mice of the same gender as assessed by a two-tailed, unpaired Student’s t-test. *, P≤0.05; **, P≤0.01; ***, P≤0.001; ****, P≤0.0001; and *****, P≤0.00001.

The 9 genes that had an altered expression in the *Cyp46a1*^*-/-*^ brain in the PCR array and Ct numbers ≤ 30 were quantified by qRT-PCR (Fig 4). These quantifications confirmed the altered mRNA levels of *Cyp46a1*, *Srebf2*, *Ldlr*, *Fdps*, *Nsdhl*, and *Apoa2* and showed no changes in the expression of *Prkag2*, *Snx17*, and *Lipe*. The highest changes were in *Cyp46a1*, whose expression was decreased 71- and 8.3-fold in female and male *Cyp46a1*^*-/-*^ mice, respectively, and in *Apoa2*, whose expression was increased 6-fold and 2.3-fold in female and male mice, respectively.

In addition, the expression of other genes was assessed. These genes were related to SREBFs (*Srebf1a*, *Scap*, and ten SREBF targets) [13, 51], LXRs (*Lxrα*, *Lxrβ*, and four LXR targets) [53], cholesterol esterification (*Acat1*) [54], and post-translational regulation of cholesterol homeostasis (*Prkaa1*, *Prkag2*, *Ubc-7*, and *Gp78*) [16, 55]. The mRNA levels of the SREBF2 target *Hmgcr*, a key enzyme in cholesterol biosynthesis, were decreased only by 18% in female mice and by 13% in male mice (Fig 4A). Similarly, there were small but statistically significant changes in many other cholesterol-biosynthetic genes ranging from 29% downregulation (*Hmgcs2* in female mice) to 46% upregulation (*Sc5d* in female mice). Only the SREBF2 target *Dhcr24* exhibited ≥2.0-fold downregulation in both *Cyp46a1*^*-/-*^ genders. However, this down-regulation did not lead to the accumulation of either desmosterol or zymosterol, the substrates of DHCR24 (Fig 2B). Collectively, moderate changes in the expression of the genes pertinent to cholesterol biosynthesis suggested that transcriptional regulation *via* SREBF2 is not significantly activated in the *Cyp46a1*^*-/-*^ brain to explain a compensatory decrease in cholesterol biosynthesis.

There was a differential effect of CYP46A1 deficiency on the mRNA brain levels of the LXR isoforms and some of their targets (Fig 4C). In the brain of wild type mice, *Lxrα* was less abundant than *Lxrβ* (the Ct values of *Lxrα* and *Lxrβ* were 28–28 and 22–24, respectively). Yet, it was *Lxrα* but not *Lxrβ* that was upregulated 2.8-fold in the female *Cyp46a1*^*-/-*^ brain. Consistent with this upregulation, the target genes *Abca1* and *Apoe* were upregulated 2.2- and 1.3-fold, respectively, as well. In the male *Cyp46a1*^*-/—*^brain, the mRNA levels for *Lxrα* and *Lxrβ* were unchanged but the *Abca1* expression was still increased 1.8-fold. The LXR activating ligands 24- and 27-hydroxycholesterol were not detected in the *Cyp46a1*^*-/-*^ brain, and the levels of desmosterol were decreased as compared to the wild type brain (Fig 2B and 2C). Thus, there should be an alternative mechanism of *Abca1* upregulation as well as the mechanism(s) to maintain the wild type level of expression of *Apoe* in the male *Cyp46a1*^*-/—*^brain and *Idol* in the female *Cyp46a1*^*-/—*^brain. Only a decreased expression of *Apod* in both genders was consistent with a lack of a strong LXR activator 24-hydroxycholesterol in the *Cyp46a1*^*-/-*^ brain.

The evaluation of changes in mRNA levels for proteins involved in post-translational control of cholesterol homeostasis documented significant increases (7.7- and 10.0-fold in female and male *Cyp46a1*^*-/-*^ mice, respectively) in the expression of *Prkaa1*, a catalytic α-subunit of AMPK, which phosphorylates and suppresses the activities of HMGCR and SREBF1c [14, 15]. There were also increases in the expression of *Ubc-7* (1.4- and 5-fold in female and male *Cyp46a1*^*-/-*^ mice, respectively) as well as *Gp78* (1.5- and 1.6-fold in female and male *Cyp46a1*^*-/-*^ mice, respectively) involved in the ubiquitination of HMGCR (*Ubc-7 and Gp78*) and INSIGSs (*Gp78*), the SREBF retention proteins. However, the mRNA levels of *Prkag2*, a noncatalytic regulatory γ-subunit of AMPK, were not changed. The *Cyp46a1*^*-/-*^ brain phosphoproteome and brain protein ubiquitination were next investigated to evaluate the significance of changes in the expression of *Prkaa1*, *Ubc-7*, and *Gp78*,

A lack of ACAT1, catalyzing cholesterol esterification in the brain, was previously shown to lead to an increase in cholesterol metabolism by CYP46A1 [9]. A reciprocal association was found to also exist, namely an increase in cholesterol esterification in the *Cyp46a1*^*-/-*^ brain (Fig 2A). Consistent with the latter finding, the brain mRNA quantifications showed a significant upregulation of *Acat1* in *Cyp46a1*^*-/-*^ mice of both genders (Fig 4D). Transcriptional regulation of ACAT1 is not a common mechanism of this enzyme regulation but was reported to be operative in macrophages and controlled by specific proteins or a small molecule [56]. 25-Hydroxycholesterol is potent allosteric activators of ACAT1 *in vitro* [57]. It is feasible that 24-hydroxycholesterol is also an ACAT1 activator, and the *Cyp46a1*^*-/-*^ brain compensates for a lack of this activator by an increase in the *Acat1* expression. This is currently only a speculation and that there is no known mechanism behind such a response.

### Protein levels of SREBF2, HMGCR, ABCA1, and HDACs

**Fig 5:**
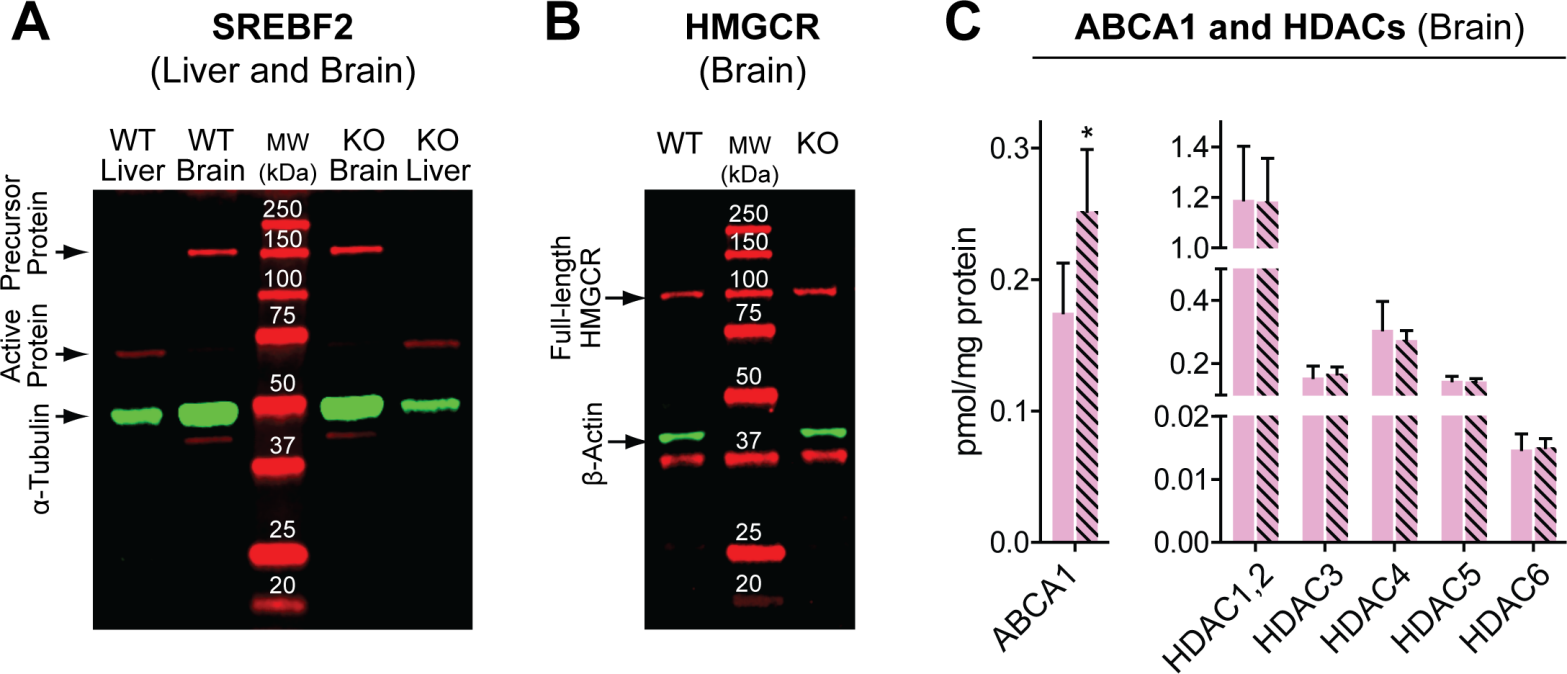
Quantification of proteins in the *Cyp46a1*^*-/-*^ brain. A) and B) Representative Western blots for SREBF2 (n = 3 female mice 3–4 months of age) and HMGCR (n = 3 male mice 6 months of age). α-Tubulin and β-actin were used for normalizations of SREBF2 and HMGCR, respectively. WT, wild type mice, KO, *Cyp46a1*^*-/-*^ mice, MW, molecular weight standards. C) Mass spectrometry quantifications of ABCA1 and HDACs. Each bar represents the mean ± SD of individual measurements in 3 female mice of 3 months of age; the color code is the same as in Fig 2. Three to four transitions per peptide (two peptides for HDAC4 and one peptide for all other isoforms) were used. Black asterisks indicate statistical significance between *Cyp46a1*^*-/-*^ and wild type mice as assessed by a two-tailed, unpaired Student’s t-test. *, P≤0.05.

These proteins were quantified in the *Cyp46a1*^*-/-*^ and wild type brains to further assess the role of transcriptional regulation of cholesterol biosynthesis and a possibility of posttranslational regulation (deacetylation) of LXRs by HDACs. Remarkably, in both wild type and *Cyp46a1*^*-/-*^ mice, SREBF2 was detected only as the precursor protein (120 kDa) but not the active form (68 kDa) (Fig 5A). In contrast, in the liver, mainly the active form was present in both genotypes, possibly because cholesterol biosynthesis is relatively slow in the brain [58] and much faster in the liver [59]. In any case, *Cyp46a1*^*-/-*^ ablation did not seem to affect the protein levels of the SREBF2 precursor protein and its active form either in the brain or liver. Similarly, a lack of CYP46A1 did not seem to affect protein expression of HMGCR, which was detected as full-length protein (~97 kDa) (Fig 5B). A band corresponding to the 36 kDa protein was present in the Western blot for HMGCR. However, the identity of this band as a possible HMGCR cleavage product was not investigated. Thus, a moderate downregulation of *Srebf2* and *Hmgcr* in the *Cyp46a1*^*-/-*^ brain (Fig 4A) did not appear to translate into the decreases at the protein level. In contrast, the protein expression of ABCA1 was 1.4-fold higher in the *Cyp46a1*^*-/-*^ brain as compared to the wild type brain (Fig 5C), consistent with the mRNA level increase (Fig 4C) and suggesting that regulation of *Abca1* could be a mechanism to maintain the steady-state cellular cholesterol levels in the absence of CYP46A1. The protein levels of the HDAC isoforms were unchanged in the *Cyp46a1*^*-/-*^brain, suggesting that these enzymes are not a part of the compensatory response elicited by *Cyp46a1* ablation.

### Brain phosphoproteome

A total of 185 phosphopeptides, each containing from 1 to 3 phosphorylated amino acid residues (serine or/and threonine), were detected that showed a statistically significant difference in abundance (P≤0.05) between the *Cyp46a1*^*-/-*^ and wild type samples of the brain (S2 Table). Of them, 151 phosphopeptides were less abundant in *Cyp46a1*^*-/-*^ mice, including one peptide (from SHANK2) that was not present in the *Cyp46a1*^*-/-*^ brain. Conversely, 34 phosphopeptides were more abundant in *Cyp46a1*^*-/-*^ mice, including one peptide (from MAP1B) that was not found in the wild type brain. The identified 185 phosphopetides were from 146 distinct proteins, 7 of which were protein kinases (LMTK2, MARK1, PAK1, PTK2B, PRKCG, SIK3, and STK32C). Also, of these 146 proteins, 20 had 2 or more phosphorylated peptides. Of them, 14 (ADCY9, ADD1, ANK2, ATP2B2, EPB4.1L1, HCN2, HDGF, IMPACT, LOC102641872, MAP2, MAPT, SMAP2, UBR4, 2310022B05RIK) had all phosphopeptides with a decreased abundance in the *Cyp46a1*^*-/-*^ brain; one protein (NEFH) had an increased phosphopeptide abundance in the *Cyp46a1*^*-/-*^ brain, and 5 proteins (MAP1A, MAP1B, MARCKS, NEFM, and OXR1) had phosphopeptides with opposite changes in phosphopeptide abundance in the *Cyp46a1*^*-/-*^ brain.

### Brain protein ubiquitination

Only 6 ubiquitinated peptides (all from distinct proteins) showed a statistically significant difference in abundance in the *Cyp46a1*^*-/-*^ brain as compared to the wild type brain (Table 2). Of these 6 peptides, four were found only in the *Cyp46a1*^*-/-*^ brain; they were from STX1B (syntaxin-1B), ALDOA (fructose-biphosphate aldolase, sodium/potassium-transporting ATPase subunit alpha), ATP1A2 (sodium/potassium-transporting ATPase subunit alpha-2), and TUBA1A (tubulin alpha-1A chain). Two peptides were present in both *Cyp46a1*^*+/+*^and *Cyp46a1*^*-/-*^ brains, at a 50.1-fold and 1.8-fold higher abundance, respectively; they were from YWHAZ (914-3-3 protein zeta/delta) and UBE2N (ubiquitin-conjugating enzyme E2N).

**Table 2:** Ubiquinated peptides with differential abundance in the *Cyp46a1*^*-/-*^ brain as compared to the wild type brain.

Protein	Peptide Sequence	Ubiquitination Site	Ratio (KO/WT)	Biological Process
STX1B	LAIFTDDIKMDSQMoTK	K9	KO only	Cognition
ATP1A2	IMoDSFKNMoVPQQALVIR	K6	KO only	Cognition, energy production
ALDOA	IVAPGKGILAADESTGSIAK	K6	KO only	Energy production
TUBA1A	VGINYQPPTVVPGGDLAKVQR	K18	KO only	Cytoskeleton function
YWHAZ	NLLSVAYKNVVGAR	K8	50.1	
UBE2N	ICLDILKDK	K7	1.8	Ubiquitination

KO, *Cyp46a1*^*-/-*^ brain; WT, wild type brain, Mo, oxidized methionine.

## Discussion

We completed here our previous sterol quantifications in the brain of *Cyp46a1*^*-/-*^ mice [49] and further characterized the *Cyp46a1*^*-/-*^ brain for gene expression and protein post-translational modifications. The brain levels of all the major sterols in the cholesterol biosynthesis pathway were decreased in *Cyp46a1*^*-/-*^ mice (Fig 2B), nevertheless the brain expression of the cholesterologenic genes was altered only moderately in these animals (Fig 4A). Moreover, the protein expression of SREBF2 and HMGCR, the two key players in cholesterol biosynthesis, was unchanged (Fig 5A and 5B), suggesting that transcriptional regulation of cholesterol biosynthesis was not significantly activated in the *Cyp46a1*^*-/-*^ brain.

Studies of the gene expression brought attention to the two compensatory responses in the *Cyp46a1*^*-/-*^ brain, which were not considered previously. The first response is a significant upregulation of *Acat1*, a cholesterol-esterifying gene (Fig 4D). This upregulation made a small increase in cholesterol esterification in the *Cyp46a1*^*-/-*^ brain (Fig 2A) more meaningful and suggested a mechanism for this increase. Cholesterol esters are stored inside cells in the form of lipid droplets. Accordingly, an increase in cholesterol esterification in the *Cyp46a1*^*-/-*^ brain represents an increase in the intracellular cholesterol storage, a compensatory mechanism to a lack of cholesterol metabolism. The second response is an upregulation of *Abca1* and *Apoa2* (Fig 4C and 4E), along with a confirmed increase in the ABCA1 protein expression (Fig 5C). This response suggests the existence of the compensatory mechanism in the *Cyp46a1*^*-/-*^ brain, which could be related to the CYP46A1-indepenent pathway of cholesterol removal. This non-CYP46A1 pathway is believed to involve both ABCA1 (a cellular cholesterol efflux transporter) and APOA1, an apolipoprotein, which carries the effluxed cholesterol [7]. No information, however, is available about the role of APOA2 in this process. It is conceivable that in the central nervous system, like in the serum, APOA2 is present on the APOA1-containing lipoprotein particles, which in the serum are formed by fusion of nascent particles containing individual APOA1 and APOA2 [60]. The proposed role of APOA2 would be consistent with a higher *Apoa2* upregulation in female than male *Cyp46a1*^*-/-*^ mice (Fig 4E), and an apparent association of this upregulation with higher decreases in cholesterol precursors' levels in female animals (Fig 2B). In the brain, ABCA1 is highly expressed in the hippocampal pyramidal neurons and cerebellar Purkinje cells [61, 62], the same type of neurons which highly express CYP46A1 [63]. It is plausible that CYP46A1-independent pathway of cholesterol removal becomes upregulated in *Cyp46a1*^*-/-*^ mice to compensate for a lack of cholesterol metabolism. In agreement with this hypothesis, a reciprocal, 5–10 fold downregulation of *Apoa1* and *Apoa2* was observed in the brain of mice with increased metabolism of cholesterol mediated by CYP46A1 [37]. The detailed mechanism behind the CYP46A1-independent cholesterol removal is currently unknown, and a possible role of APOA2 in this process is a new hypothesis that we propose.

No phosphorylated peptides either from HMGCR, SREBF or any other cholesterol biosynthetic proteins were detected in our study of the brain phosphoproteome, possibly because the phosphopeptides from the proteins of our interest were of a low abundance. Thus, the question about the major mechanism of cholesterol biosynthesis regulation remains open. An important finding was a lowered (~2.5-fold) phosphorylation of RAB3IP (Rab-3A-interacting protein), which could provide an explanation for an increase in LXR activity in the absence (or significant reduction) of the activating ligands such as 24-hydroxycholesterol and desmosterol. Evidence was presented that CYP46A1 overexpression inhibits LXR activity by activating small GTPases (guanosine triphosphate-binding proteins), including RAB, through their increased prenylation [64]. RAB3IP (or Rabin8) is a guanine nucleotide exchange factor and a major activator of RAB8 sGTPases in cells [65, 66] after phosphorylation relieves the autoinhibition of RAB3IP [67]. The *Cyp46a1*^*-/-*^ brain had a decreased phosphorylation of the same peptide whose increased phosphorylation relieves RAB3IP autoinhibition (S2 Table). Hence, RAB8 may not be activated in the *Cyp46a1*^*-/-*^ brain and inhibit LXR, a potential mechanism for the LXR-dependent *Abca1* upregulation and the wild type-level *Apoe* expression (Fig 4C). Also, RAB8 is vital for neurite outgrowth and regulates vesicular traffic from the *trans*-Golgi to the plasma membranes including AMPA receptor trafficking [68, 69]. A reduction in RAB3IP phosphorylation and putative effect on RAB8 suggests that dendritic outgrowth could be decreased in the *Cyp46a1*^*-/-*^ brain as well, consistent with an opposite effect, an increase in dendritic outgrowth, observed upon CYP46A1 overexpression [70]. Further studies are required to confirm the RAB8-mediated mechanisms in the *Cyp46a1*^*-/-*^ brain and whether they are affected by phosphorylation and prenylation.

Besides RAB8, two other members of the sGTPase superfamily of proteins (CDC42 and RAC), could be affected in the *Cyp46a1*^*-/-*^ brain as indicated by a ~5-fold decrease in a phosphorylation of PAK1. PAK1 is a protein kinase which is autophosphorylated and has an increased activity toward different substrates after interaction with activated CDC42 and RAC [71]. The substrates of PAK1 are multiple and affect a wide range of cellular pathways (e.g., MAPK-, AKT-, WNT1/β-catenin-, ERα-, BAD-, and NF-κB-dependent) as well as processes (e.g., cytoskeleton remodeling, mitotic events, and gene expression) [72]. CDC42 was reported to be activated by a cholesterol reduction during LTP and mediate the AMPA receptor delivery to the synaptic membrane from RAB11-recycling endosomes [73]. *Cyp46a1*^*-/-*^ mice have impaired learning and hippocampal LTP [41, 42], and hence could have a reduced CDC42 activation and impaired AMPA receptor synaptic delivery. If so, a decreased phosphorylation of both PAK1 and RAB3IP in *Cyp46a1*^*-/-*^ mice could underlie impaired AMPA receptor synaptic delivery.

In addition to RAB3IP and PAK1, a lack of CYP46A1 affected the phosphorylation of many other proteins and protein kinases, which were not apparently linked. This suggests that CYP46A1-mediated cholesterol elimination exerts a general effect on protein phosphorylation, which could relate to membrane cholesterol and receptor molecules associated with cholesterol-rich lipid rafts. Indeed, cholesterol loss was shown to enhance TRKB (tropomyosin receptor kinase B) signaling [74, 75], which activates three main intracellular signaling cascades: the Ras–mitogen-activated protein kinase (MAPK) pathway, the phosphatidylinositol 3-kinase (PI3K)–Akt pathway, and the phospholipase Cγ (PLCγ)–Ca^2+^ pathway [76]. One of these pathways, PI3K, leads to the phosphorylation of MAP1B (microtubule-associated protein 1B), which was found to be affected in the present work (S2 Table). MAP1B has 33 phosphorylation sites modified by different protein kinases, and the phosphorylation of these sites has different effects on protein function [77]. In the *Cyp46a1*^*-/-*^ brain, MAP1B had the highest number of the identified phosphorylated peptides with differential abundance as compared to the wild type brain (a total of 10), and the phosphorylation of these peptides was both decreased and increased (S2 Table). A similar pattern of changes in the phosphopeptide abundance was observed for MAP1A, another member of the MAP family, whereas the phosphorylation of the peptides from MAP2, MAPT and MAP6, also belonging to the MAP family, was only decreased in the *Cyp46a1*^*-/-*^ brain. The MAP family is phosphorylated by microtubule-affinity-regulating-kinases (MARKs) [78]; one of these isoforms, MARK1 had a decreased phosphorylation in the *Cyp46a1*^*-/-*^ brain. In addition, MAPT can be phosphorylated by protein kinase C (PRKC) [78], one of whose isoforms (PRKCG, the gamma type of PRKC) was also less phosphorylated in the *Cyp46a1*^*-/-*^ brain. Finally, MAPT can be phosphorylated by MAPK [78] activated by TRKB, whose function depends on membrane cholesterol [74, 75]. The alternative splicing of MAPT produces tau proteins that play key roles in regulating microtubule dynamics, axonal transport, and neurite outgrowth [79]. These functions are regulated by tau phosphorylation at specific sites, which if disrupted, leads to tau dysfunction and tau pathology [79]. In addition to tau, neuronal cytoskeletal proteins are represented by NEF (neurofilament) proteins, which are hyperphosphorylated in Alzheimer's disease [80]. All three of the identified phosphopeptides from the heavy polypeptide of NEF (NEFH) and one phosphopeptide from the medium polypeptide of NEF (NEFM) were more phosphorylated in the *Cyp46a1*^*-/-*^ brain than the wild type brain; one phosphopeptide from NEFM was less phosphorylated in the *Cyp46a1*^*-/-*^ brain (S2 Table). Previously, CYP46A1 deficiency or increased enzyme activity were mainly considered in the context of the amyloid β pathology [9, 37, 81]. The data of the present work along with the results on CYP46A1 inhibition in a mouse model of Alzheimer's disease [82] highlight a new link between the CYP46A1 deficiency and Alzheimer’s disease, namely abnormal phosphorylation of neuronal cytoskeletal proteins.

Cognitive dysfunction, including impairments in spatial, associative, and motor learning, is a hallmark of the *Cyp46a1*^*-/-*^ genotype, which results, at least in part, from impaired LTP due to decreased protein prenylation [41, 42]. Our data suggest that protein phosphorylation could be another mechanism responsible for the *Cyp46a1*^*-/-*^ cognitive phenotype, and in addition, affects other processes in the brain pertinent to cognition (Table 3).

**Table 3:** Phosphoproteins with different phosphopeptide abundance in the *Cyp46a1*^*-/-*^ brain as compared to the wild type brain.

*Abnormal Spatial Learning*	*Abnormal Motor Learning*	*Impaired Ability to Fire Action Potentials*	*Abnormal Neurotransmitter Secretion*	*Abnormal Synaptic Vesicle Clustering*
ADD2 (**↓**)	HCN1 (**↓**)	KCNA2 (**↓**)	STXBP1 (**↓**)	CTNNB1 ()
AMPH (**↓**)	MAPT (**↓**)	KCNMA1 (**↓**)	SYN1 (**↓**)	KIF1A (**↓**)
ATP1A2 ()	SHANK3 (**↓**)	MADD **()**	SYT1 **()**	SYN1 (**↓**)
CTNND2 (**↓**)	SLC24A2 (**↓**)	***Increased Synaptic Depression***	***Abnormal Synaptic Vesicle Number***	***Abnormal Synaptic Transmission***
HCN1 (**↓**)	***Reduced Long Term Potentiation***	KCNMA1 (**↓**)	BSN (**↓**)	BSN (**↓**)
MAPT (**↓**)	ADD2 (**↓**)	SYN1 (**↓**)	DNAJC6 (**↓**)	SHANK3 (**↓**)
NCAM1 (**↓**)	CCDC88A (**↓**)	SYT1 **()**	KIF1A (**↓**)	STXBP1 (**↓**)
PRKCG (**↓**)	CTNNB1 **()**	***Abnormal Neuron Physiology***	MADD **()**	SYT1 **()**
SHANK2 (**↓**)	MAPT (**↓**)	**HCN2** (**↓**)	MAP6 (**↓**)	***Abnormal Nervous System Electrophysiology***
SHANK3 (**↓**)	PRKCG (**↓**)	KCNQ2 (**↓**)	PCDH17 (**↓**)	ATP1A2 ()
SLC24A2 (**↓**)	SHANK2 (**↓**)	SHANK3 (**↓**)	***Abnormal Synaptic Vesicle Recycling***	HCN1 (**↓**)
SLC8A2 (**↓**)	SHANK3 (**↓**)	SLC8A2 (**↓**)	AMPH (**↓**)	**HCN2 (↓)**
***Abnormal Long Term Object Recognition Memory***	SLC24A2 (**↓**)	SYT1 **()**	DNAJC6 (**↓**)	KCNA2 (**↓**)
BRAF (**↓**)	***Abnormal Long Term Depression***	***Abnormal Axon Morphology***	SYN1 (**↓**)	PLCL1 (**↓**)
CCDC88A (**↓**)	SHANK3 (**↓**)	**ANK2 (↓)**	***Abnormal Excitatory Postsynaptic Currents***	***Axon Degeneration***
MAPT (**↓**)	SLC24A2 (**↓**)	DST (**↓**)	CCDC88A (**↓**)	DST (**↓**)
SHANK3 (**↓**)	SLC8A2 (**↓**)		MADD **()**	**KIF1A** (**↓**)
***Abnormal Contextual Conditioning Behavior***	***Abnormal Cerebellum Morphology***	GAP43 (**↓**)	PRKCG (**↓**)	**NEFH ()**
ADD2 (**↓**)	*** ***	**MAP1B (↓)**	SHANK3 (**↓**)	STXBP1 (**↓**)
AMPH (**↓**)	**ATP2B2 (↓)**	MAPT (**↓**)	SLC1A3 (**↓**)	
CTNND2 (**↓**)	CIT (**↓**)	**NEFH ()**	SYT1 **()**	
**MAP2 (↓)**	**MAP1B (←↓)**	PLEC (**↓**)		
MAPT (**↓**)	MYO5A (**↓**)			
PRKCG (**↓**)	NCAM1 (**↓**)			

Phosphoproteins were grouped by the GeneAnalytics (LifeMap Sciences) software and are shown based on involvement in brain processes pertinent to cognition.

**↑** or **↓** indicate an increase or decrease, respectively, in the phosphopeptide abundance.

Proteins in bold are those with 2 or more phosphopeptides with differential abundance in the *Cyp46a1*^*-/-*^ brain as compared to the wild type brain.

For example, LTP along with long term depression (LTD) and excitatory postsynaptic currents specifically require PRKCG (cGMP-dependent protein kinase), whose phosphorylation was decreased ~3-fold in the *Cyp46a1*^*-/-*^ brain [83]. LTP also requires MAPT, whose phosphorylation was decreased in the *Cyp46a1*^*-/-*^ brain as was the phosphorylation of SHANKs (SH3 and multiple ankyrin repeat domains proteins), the scaffold proteins at excitatory synapses in the CNS which interact with multiple partners and are crucial for proper synaptic development and function [84]. In the *Cyp46a1*^*-/-*^ brain, phosphorylated SHANK2 was not even detected, and the phosphorylation of SHANK3 was decreased almost 2-fold (S2 Table). Phosphorylation is important for SHANK3 function and is required for the downstream Erk-MAPK and PI3K signaling [85]. Another scaffold protein affected in the *Cyp46a1*^*-/-*^ brain is BSN (bassoon presynaptic cytomatrix protein), a key player in structural organization and functional regulation of presynaptic release sites [86]. BSN is one of the most heavily phosphorylated synaptic proteins, and its phosphorylation is implied in the regulation of neurotransmitter release [77, 87, 88]; ~7-fold decrease in BSN phosphorylation in the *Cyp46a1*^*-/-*^ brain may be relevant to impaired synaptic transmission. SLCs (solute carriers) are a big group of membrane proteins which transport different solutes including glutamate and amino acids (SLC1A3) or serve as the Na^+^/Ca^2+^ (SLC8A2) and Na^+^/(Ca^2+-^K^+^) (SLC24A2) exchangers [89]. The phosphorylation of all the detected members of the SLC family was decreased in the *Cyp46a1*^*-/-*^ brain (S2 Table), and these members are not only involved in LTP but also in LTD and neuron physiology including excitatory postsynaptic currents (Table 3). Thus, the phosphorylation of a number of proteins involved in synaptic transmission was altered in the *Cyp46a1*^*-/-*^ brain.

Ubiquitination is an important post-translational modification that alters protein stability, localization or interaction property [90]. The ubiquitination of only a limited number of proteins seemed to be affected in the *Cyp46a1*^*-/-*^ brain, and the extent of ubiquitination was always increased (Table 2). The affected proteins were of pertinence to ubiquitination *per se* (UBE2N), cognition (STX1B and ATP1A2), cytoskeleton function (TUBA1A and YWHAZ), and energy production (ATP1A2 and ALDOA). On the basis of the known protein functions [91–99], increased ubiquitination of all the affected proteins in the *Cyp46a1*^*-/-*^ brain could relate, directly or indirectly, to the observed cognitive deficits in *Cyp46a1*^*-/-*^ mice; of course, the precise effect (if any) of this post-translational modification on the *Cyp46a1*^*-/-*^ brain needs to be further investigated.

In conclusion, the evaluations of the *Cyp46a1*^*-/-*^ brain brought attention to the previously unconsidered compensatory responses, pathways, and proteins that may underlie cognitive deficits in *Cyp46a1*^*-/-*^ mice. The data obtained enhance our understanding of CYP46A1 and the role of cholesterol metabolism in the brain.

## Supporting information

S1 Text The abbreviations used in the present work. Gene symbols are italicized and begin with an uppercase letter; protein symbols have all letters in uppercase. If gene and protein symbols have the same abbreviation, only one that is mentioned first is given below.
(PDF)

S1 Table Primers for qRT-PCR. (PDF)

S2 Table Alphabetical list of proteins and phosphopeptides with differential abundance in the *Cyp46a1*^*-/-*^ brain as compared to the wild type brain. Proteins with several identified phosphopeptides are shown first followed by the proteins with only one identified phosphopeptide. WT, wild type mice; KO, *Cyp46a1*^*-/-*^ mice; Mo, oxidized methionine. The KO/WT peptide ratios higher than 1 are in bold.
(PDF)
