# Correction: Transcriptional and post-translational changes in the brain of mice deficient in cholesterol removal mediated by cytochrome P450 46A1 (CYP46A1)

**DOI:** 10.1371/journal.pone.0191058

**Published:** 2018-01-05

**Authors:** The PLOS ONE Staff

There are errors in Table 3. Some of the arrows are missing in the published table. The publisher apologizes for this error. Please see the corrected Table 3 here.

**Table 3:** Phosphoproteins with different phosphopeptide abundance in the *Cyp46a1*^*-/-*^ brain as compared to the wild type brain.

***Abnormal Spatial Learning*** ADD2 (**↓**) AMPH (**↓**) ATP1A2 (↑) CTNND2 (**↓**) HCN1 (**↓**) MAPT (**↓**) NCAM1 (**↓**) PRKCG (**↓**) SHANK2 (**↓**) SHANK3 (**↓**) SLC24A2 (**↓**) SLC8A2 (**↓**) ***Abnormal Long Term Object Recognition Memory*** BRAF (**↓**) CCDC88A (**↓**) MAPT (**↓**) SHANK3 (**↓**) ***Abnormal Contextual Conditioning Behavior*** ADD2 (**↓**) AMPH (**↓**) CTNND2 (**↓**) **MAP2 (↓)** MAPT (**↓**) PRKCG (**↓**)	***Abnormal Motor Learning*** HCN1 (**↓**) MAPT (**↓**) SHANK3 (**↓**) SLC24A2 (**↓**) ***Reduced Long Term Potentiation*** ADD2 (**↓**) CCDC88A (**↓**) CTNNB1 **(↑)** MAPT (**↓**) PRKCG (**↓**) SHANK2 (**↓**) SHANK3 (**↓**) SLC24A2 (**↓**) ***Abnormal Long Term Depression*** SHANK3 (**↓**) SLC24A2 (**↓**) SLC8A2 (**↓**) ***Abnormal Cerebellum Morphology*** **ATP2B2 (↓)** CIT (**↓**) **MAP1B (↑↓)** MYO5A (**↓**) NCAM1 (**↓**)	***Impaired Ability to Fire Action Potentials*** KCNA2 (**↓**) KCNMA1 (**↓**) MADD **(↑)** ***Increased Synaptic Depression*** KCNMA1 (**↓**) SYN1 (**↓**) SYT1 **(↑)** ***Abnormal Neuron Physiology*** **HCN2** (↓) KCNQ2 (**↓**) SHANK3 (**↓**) SLC8A2 (**↓**) SYT1 **(↑)** ***Abnormal Axon Morphology*** **ANK2 (↓)** DST (**↓**) GAP43 (**↓**) **MAP1B (↑↓)** MAPT (**↓**) **NEFH (↑)** PLEC (**↓**)	***Abnormal Neurotransmitter Secretion*** STXBP1 (**↓**) SYN1 (**↓**) SYT1 **(↑)** ***Abnormal Synaptic Vesicle Number*** BSN (**↓**) DNAJC6 (**↓**) KIF1A (↓) MADD **(↑)** MAP6 (↓) PCDH17 (**↓**) ***Abnormal Synaptic Vesicle Recycling*** AMPH (**↓**) DNAJC6 (↓) SYN1 (**↓**) ***Abnormal Excitatory Postsynaptic Currents*** CCDC88A (**↓**) MADD **(↑)** PRKCG (**↓**) SHANK3 (**↓**) SLC1A3 (**↓**) SYT1 **(↑)**	***Abnormal Synaptic Vesicle Clustering*** CTNNB1 (↑) KIF1A (**↓**) SYN1 (**↓**) ***Abnormal Synaptic Transmission*** BSN (**↓**) SHANK3 (**↓**) STXBP1 (**↓**) SYT1 **(↑)** ***Abnormal Nervous System Electrophysiology*** ATP1A2 (↑) HCN1 (**↓**) **HCN2 (↓)** KCNA2 (**↓**) PLCL1 (**↓**) ***Axon Degeneration*** DST (↓) **KIF1A** (**↓**) **NEFH (↑)** STXBP1 (**↓**)

Phosphoproteins were grouped by the GeneAnalytics (LifeMap Sciences) software and are shown based on involvement in brain processes pertinent to cognition.

**↑** or **↓** indicate an increase or decrease, respectively, in the phosphopeptide abundance.

Proteins in bold are those with 2 or more phosphopeptides with differential abundance in the *Cyp46a1*^*-/-*^ brain as compared to the wild type brain.
