# Supplementary material for: Transcriptional and post-translational changes in the brain of mice deficient in cholesterol removal mediated by cytochrome P450 46A1 (CYP46A1)
Source: PLoS One. 2017 Oct 26;12(10):e0187168. doi: 10.1371/journal.pone.0187168 (PMC5658173; doi:10.1371/journal.pone.0187168)
Supplement: S1 Text — Gene symbols are italicized and begin with an uppercase letter; protein symbols have all letters in uppercase. If gene and protein symbols have the same abbreviation, only one that is mentioned first is given below. (PDF) [file pone.0187168.s001.pdf]

S1 Text. The abbreviations used in the present work. Gene symbols are italicized and begin with an uppercase letter; protein symbols have all letters in uppercase. If gene and protein symbols have the same abbreviation, only one that is mentioned first is given below.

Gene Abbreviations—Akr1d1 (aldo-keto reductase family 1 member D1), *Apoa4* (apolipoprotein A4), *Apoc3* (apolipoprotein C3), *Cela3b* (chymotrypsin like elastase family member 3B), *Cyp51* (cytochrome P450 family 51), *Fdps* (farnesyl diphosphate synthase), *Il4* (interleukin 4), *Lipe* (lipase E, hormone sensitive type), *Npc1l1* (NPC1 like intracellular cholesterol transporter 1), *Nr0b2* (nuclear receptor subfamily 0 group B member 2), *Prkaa1* (protein kinase AMP-activated catalytic subunit alpha 1), *Prkag2* (protein kinase AMP-activated non-catalytic subunit gamma 2), *Snx17* (sorting nexin 17), and *Ubc-7* (ubiquitin conjugating enzyme E2 G1).

Protein Abbreviations—2310022B05RIK (uncharacterized protein C1orf198 homolog), ABCA1 (ATP binding cassette subfamily A member 1), ACAT1 (acyl-coenzyme A:cholesterol acyltransferase 1), ADCY9 (adenylate cyclase type 9), ADD1 (alpha-adducin), AKT (RAC-alpha serine/threonine-protein kinase), ALDOA (fructose-bisphosphate aldolase A), AMPK (AMP-activated protein kinase), ANK2 (ankyrin-2), APOA1 (apolipoprotein A1), APOA2 (apolipoprotein A2), APOD (apolipoprotein D), APOE (apolipoprotein E), APOJ (apolipoprotein J), ATP1A2 (sodium/potassium-transporting ATPase subunit alpha-2), ATP2B2 (plasma membrane calcium-transporting ATPase 2), BAD (Bcl2-associated agonist of cell death), BSN (bassoon presynaptic cytomatrix protein), CDC42 (cell division control protein 42 homolog), CYP11A1 (cytochrome P450 family 1 subfamily A member 1), CYP27A1 (cytochrome P450 family 27 subfamily A member 1) ,CYP46A1 (cytochrome P450 family 46 subfamily A member 1), DHCR24 (delta(24)-sterol reductase), EBP (emopamil binding protein), EPB4.1L1 (band 4.1-like protein 1), ERα (nuclear receptor subfamily 3 group A member 1), FBW7 (F-box/WD repeat-containing protein 7), GP78 (E3 ubiquitin-protein ligase AMFR), HCN2 (potassium/sodium hyperpolarization-activated cyclic nucleotide-gated channel 2), HDAC (histone deacetylase), HDGF (hepatoma-derived growth factor), HMGCR (3-hydroxy-3-methyl-glutaryl-coenzyme A reductase), HMGCS2 (3-hydroxy-3-methylglutaryl-CoA synthase 2), HRD1 (E3 ubiquitin-protein ligase synoviolin), HSD17B7 (hydroxysteroid 17-beta dehydrogenase 7), IDOL (inducible degrader of the LDL-receptor), IMPACT (imprinted and ancient gene protein), INSIG (insulin-induced gene 1 protein), LDLR (low-density lipoprotein receptor), LMTK2 (serine/threonine-protein kinase LMTK2), LOC102641872 (trafficking protein particle complex subunit 10), LRP (lipoprotein receptor-related protein), LXRα (liver X receptor alpha), LXRβ (liver X receptor beta), MAP1A (microtubule-associated protein 1A), MAP1B (microtubule-associated protein 1B), MAP2 (microtubule-associated protein 2), MAP6 (microtubule-associated protein 6), MAPK (mitogen-activated protein kinase), MAPT (microtubule-associated protein tau), MARCKS (myristoylated alanine rich protein kinase C substrate), MARK1 (MAP/microtubule affinity-regulating kinase), NEFH (neurofilament heavy polypeptide), NEFM (neurofilament medium polypeptide), NF-κB (nuclear factor kappa-light-chain-enhancer of activated B cells), NSDHL (NAD(P) dependent steroid dehydrogenase-like), OXR1 (oxidation resistance protein 1), PAK1 (p21-activated kinase 1), PI3K (phosphatidylinositol-4,5-bisphosphate 3-kinase), PLCγ (phosphoinositide phospholipase C gamma), PRKC (protein kinase C), PRKCG (protein kinase C gamma), PTK2B (protein-tyrosine kinase 2-beta), RAB3IP (Rab-3A-interacting protein), RAB8 (Ras-related protein Rab-8), RAB11 (Ras-related protein Rab-11), RAC (RAC-alpha serine/threonine-protein kinase), SCAP (sterol regulatory element-binding protein cleavage-

activating protein), SC5D*S* (sterol-C5-desaturase)*,* SHANK2 (SH3 and multiple ankyrin repeat domains protein 2), SHANK3 (SH3 and multiple ankyrin repeat domains protein 3), SIK3 (salt-inducible kinase 3), SLC1A3 (solute carrier family 1 member 3), SLC24A2 (solute carrier family 24 member 2), SLC8A2 (solute carrier family 8 member A2), SMAP2 (stromal membrane-associated protein 2), SREBF1 (sterol regulatory element binding transcription factor 1)*,* SREBF2 (sterol regulatory element binding transcription factor 2)*,* STK32C (serine/threonine-protein kinase 32C), STX1B (syntaxin-1B), SYN1 (synapsin 1), TRC8 (translocation in renal carcinoma on chromosome 8 protein), TRK (tropomyosin-related kinase), TRKB (tropomyosin receptor kinase B), TUBA1A (tubulin alpha-1A chain), UBE2N (ubiquitin-conjugating enzyme E2N), UBR4 (E3 ubiquitin-protein ligase UBR4), WNT1 (proto-oncogene Wnt-1),YWHAZ (14-3-3 protein zeta/delta).

Other Abbreviations— AMPA (α-amino-3-hydroxy-5-methyl-4-isoxazolepropionic acid), ER (endoplasmic reticulum), ERK (extracellular signal–regulated kinases), LTP (long term potentiation), LTD (long term depression), MRM (multiple reaction monitoring), PBS (phosphate-buffered saline).
