# Supplementary material for: Transcriptional and post-translational changes in the brain of mice deficient in cholesterol removal mediated by cytochrome P450 46A1 (CYP46A1)
Source: PLoS One. 2017 Oct 26;12(10):e0187168. doi: 10.1371/journal.pone.0187168 (PMC5658173; doi:10.1371/journal.pone.0187168)
Supplement: S1 Table — (PDF) [file pone.0187168.s002.pdf]

S1 Table. Primers for qRT-PCR.

Gene	Primers
Abca1	Forward: 5'-AGGCCGCACCATTATTTTGTC<br>Reverse: 5'-GGCAATTCTGTCCCCAAGGAT
Acat1	Forward: 5'-<br>CAGGAAGTAAGATGCCTGGAAC
Apoa2	Forward: 5'-CTGACCTGACAAGGGGTGTC<br>Reverse: 5'-ATGGCAAAGATTTGGTGGAG
Apod	Forward: 5'-TGAAGCCAAACAGAGCAACGT<br>Reverse: 5'-GGCATCAACGGGAAGAACTG
Apoe	Forward: 5'-GGCCCAGGAGAATCAATGAG<br>Reverse: 5'-CCTGGCTGGATATGGATGTTG
Cyp46a1	Forward: 5’-CCTTCTT CATTGCTGGTCACG<br>Reverse: 5’-TCCATCACTGTGAACGCCAAG
Cyp51	Forward: 5'-<br>AGAGCCCATCGAGAGATCAAGA
Dhcr24	Forward: 5'-CATCGTCCCACAAGTATG<br>Reverse: 5'-CTCTACGTCGTCCGTCA
Ebp	Forward: 5'-TATCGGCTATCTCCCTTGGA<br>Reverse: 5'-ATCGAGGGCTGGTTCTCTCT
Fdps	Forward: 5'-ATACCAGCAGATCTGTCCCC<br>Reverse: 5'-TGGGCTGGTGTGTAGAACTG
Gp78	Forward: 5'-<br>ACAAAGACCTATCTGAAACGTCC
Hmgcr	Forward: 5'-TTGGTCCTTGTTCACGCTCAT<br>Reverse: 5'-TTCGTCCAGACCCAAGGAAAC
Hmgcs2	Forward: 5'-TCATTGAACATCAACCGAGC<br>Reverse: 5'-GAAACAACCAGCCTTTCACC
Hsd17b7	Forward: 5'-<br>TGCTGGAATCCTGCCTAATCCACA
Idol	Forward: 5'-GGAGCATGTCCAGCACGTCTA<br>Reverse: 5'-GTGCAGGACGCATCAGATGA
Ldlr	Forward: 5'-ACCTGCCGACCTGATGAATTC<br>Reverse: 5'-GCAGTCATGTTCACGGTCACA
Lipe	Forward: 5'-CCTGTCTCGTTGCGTTTGTA<br>Reverse: 5'-ACGCTACACAAAGGCTGCTT
Lxrα	Forward: 5'-AGCGTCCATTCAGAGCAAGTG<br>Reverse: 5'-CACTCGTGGACATCCCAGATCT
Lxrβ	Forward: 5'-ACTCGGAGCAGGTCTTTGCAT<br>Reverse: 5'-CCTACTCGTGCACATCCCAGAT

Nsdhl	Forward: 5'-TCGAAGCAAAATATGGTCCC<br>Reverse: 5'-TGGAGTTCAGTGGGTGCAG
Prkaa1	Forward: 5'-GTCAAAGCCGACCCAATGATA<br>Reverse: 5'-
Prkag2	Forward: 5'-GGTGTTGACGGAGAAGAGGA<br>Reverse: 5'-TCATCCAAAGAGTCTTCGCC
Sc5d	Forward: 5'-TGGCCCGAGGACAACA<br>Reverse: 5'-GCACCCAGGTTTGTGACAA
Scap	Forward: 5'-CGTGGCGTACATCCAACAGA<br>Reverse: 5'-GTTGGAATGCTCGGGACAGA
Snx17	Forward: 5'-CAGGGGTCAAAGAGAACAGC<br>Reverse: 5'-GTGAATGGAGTCCTGCACTG
Srebf1a	Forward: 5'-ATGGACGAGCCACCCTTCA<br>Reverse: 5'-
Srebf2	Forward: 5'-ATGATCACCCCGACGTTCAG<br>Reverse: 5'-GGTCGCTGCGTTCTGGTATATC
Ubc-7	Forward: 5'-<br>CTGGCAGAACTCAACAAAAATCC
