# Supplementary material for: Transcriptional and post-translational changes in the brain of mice deficient in cholesterol removal mediated by cytochrome P450 46A1 (CYP46A1)
Source: PLoS One. 2017 Oct 26;12(10):e0187168. doi: 10.1371/journal.pone.0187168 (PMC5658173; doi:10.1371/journal.pone.0187168)
Supplement: S2 Table — Proteins with several identified phosphopeptides are shown first followed by the proteins with only one identified phosphopeptide. WT, wild type mice; KO, Cyp46a1-/- mice; Mo, oxidized methionine. The KO/WT peptide ratios higher than 1 are in bold. (PDF) [file pone.0187168.s003.pdf]

S2 Table. Alphabetical list of proteins and phosphopeptides with differential abundance in the *Cyp46a1$^{-/-}$* brain as compared to the wild type brain. Proteins with several identified phosphopeptides are shown first followed by the proteins with only one identified phosphopeptide. WT, wild type mice; KO, *Cyp46a1$^{-/-}$* mice; Mo, oxidized methionine. The KO/WT peptide ratios higher than 1 are in bold.

#	Protein	Sequence	Site of phosphorylation	Peptide ratio, KO/WT
1	ADCY9	AIEKDSCEDIGVEEASELSK	S6	0.46
		GQGTASPGSVSDLAQTVK	S6; S9	0.21
2	ADD1	EKSPPDQSAVPNTPPSTPVKLEEDLPQEPTSR	S3; S8; S16	0.53
		YSDVEVPASVTGHSFASDGDSGTCSPLR	S25	0.18
		TPSFLKK	S3	0.04
3	ANK2	KGSSEESVDEDRGLVPEPLPTAK	S3; S4; S7	0.65
		TAEGTEPKPQGAIRSPQGLELPLPNR	S15	0.30
		TEGDSPAAALSPQMHQEPVQQDFSGK	S5; S11	0.10
4	ATP2B2	SATSSSPGSPIHSLETSL	S17	0.41
		SATSSSPGSPLHSLETSL	S5; S6; S9	0.38
5	EPB4.1L1	ADSSDETDTSFAER	S3; S4	0.62
		DLGFVQLQPPGDLASPK	S15	0.18
		GPSSQEDESGGLEDSPDRGACSTPEMPQFESVK	S15	0.14
6	HCN2	DSASPGAASGLDPLDSAR	S4	0.41
		RAPPGPLPPAASPGPPAASPPAAPSSPR	S12; S19; S25	0.14
7	HDGF	AGDVLEDSPK	S8	0.34
		AGDVLEDSPKRPK	S8	0.13
8	IMPACT	KTEEVEVESEEDPILEHPPENPVK	S9	0.43
		TEEVEVESEEDPILEHPPENPVK	S8	0.26
9	LOC102641872	QESGSSLEPPSGLALEDGAHVLR	S3	0.35
		RQESGSSLEPPSGLALEDGAHVLR	S4	0.04
10	MAP1A	AELEEMoEEVHPSDEEEEETKAESFYQK	S12	**3.04**
		SPPCEDFSVTGESEK	S1	**2.42**
		EAELTPENIAAAR	T5	**2.20**
		ALGLEESPEEEGK	S7	**1.88**
		EMTLDQKSPEK	S8	0.22
		SSLLLDTVTSIPSSR	S10; S13; S14	0.11
11	MAP1B	VLSPLRSPPLLGSESPYEDFLSADSK	S7	**in KO only**
		DYNASASTISPPSSMoEEDK	S10	**10.04**
		GEAEQSEEEGEEEDKAEDAR	S6	**2.68**
		DYNASASTISPPSSMoEEDKFSK	T8	**2.61**
		FEDEGAGFEESSETGDYEEK	S12	**2.60**
		RESVVSGDDRAEEDMoDDVLEK	S3; S6	**2.13**
		TPEEGGYSYEISEK	T1	**1.65**

		VQSLEGEKLSPKSDISPLTPR	S10; S13; S16	0.62
		DVSDERLSPAKSPSLSPSPPSPIEK	S8; S12; S14	0.33
		DYNASASTISPPSSMEEDKFSK	S10; S13	0.17
12	MAP2	GLSSVPEVAEVEPTTK	S4	0.36
		DKVTDGISKSPEKR	S10	0.14
13	MAPT	TTPSPKTPPGSGEPPK	S4; S11	0.76
		AKTDHGAEIVYKSPVVSGDTSPR	S13; S17; S21	0.59
		STPTAEDVTAPLVDER	S1	0.52
		SPVVSGDTSPR	S5; S9	0.41
		SPVVSGDTSPR	S9	0.20
14	MARCKS	GEATAERPGEAAVASSPSK	S16	**1.71**
		VNGDASPAAAEPGAK	S6	0.30
15	NEFH	AKSPVKEDIKPPAEAKSPEK	S3; S17	**1.70**
		SPAEAKSPIEVKSPEK	S1; S7; S13	**1.47**
		SPAEAKSPIEVK	S1; S7	**1.22**
16	NEFM	EEAEEKEEEPEAEKSPVKSPEAK	S15; S19	**2.08**
		VSGSPSSGFR	S4	0.26
17	OXR1	RDSETEVEELR	S3	**1.37**
		VVSSTSEEEEAFTEK	S4; S6	0.46
18	SMAP2	AVGSMPTAGSAGSVPENLNLFPEPGSK	S13	0.33
		KAVGSMPTAGSAGSVPENLNLFPEPGSK	T8	0.06
19	UBR4	HVTLPSSPR	S7	0.55
		TLSDVEDQKELASPVSPELR	S13; S16	0.11
20	2310022B05RIK	SSSLDALGPAR	S3	0.60
		QSGTPPGMoGAPGIPGASIVGGAGGR	T4	0.48
21	ADD2	STPASPVQSPSK	S5; S9	0.38
22	AMPH	AFSIQGAPSDSGPLR	S11	0.31
23	ANKRD13D	GPESPQKTPPSPAPPSFEEQLR	S4; S11	0.21
24	ANO8	RPGPSPDGLLEEGSPTMVEK	S14	0.27
25	AP1M2	KQSVANGVEISVPVPSDADSPRFKTSVGSAK	S11; S16	0.64
26	AP3B2	AFYGSEEDEAK	S5	**1.29**
27	ASAP1	QASEDSNDLTPTLPETPVPLPR	S3	0.68
28	ATP1A2	VAEIPFNSTNK	T9	**2.68**
29	ATP2B3	SGSFQGAVR	S3	0.10
30	BNIP3L	DHSSQSEEEVVEGEKEVEALKK	S3; S4; S6	0.39
31	BOD1L	AVHEFLATLNHK	T8	0.15
32	BRAF	SSSAPNVHINTIEPVNIDEK	S3	0.68
33	BSN	SKTPPSNLSPIEDASPTEELR	S9; S15	0.14
34	C2CD2L	SDISERPSVDDVESETGSTGALETR	S8; S14	0.11
35	CACNA1E	EVSPMSAPNMPSIER	S3	0.18
36	CAND1	DSSSTNLESMDTS	S2	0.21

37	CCDC88A	DGVHFLPHASSSAQSPCGSPGMK	S15	0.39
38	CEP170B	SPSLGNVPNTPASTISAR	S3	**1.83**
39	CHGB	EGVDDQESLRPSNQQASK	S17	**1.86**
40	CIT	SPEHQPSAMSLLAPPSSR	S10	0.61
41	CLDN11	FYYSSGSSSPTHAK	S8; S9	**1.17**
42	CNNM1	GTPQTPKDDPVLTLLSNR	T2; T5	0.64
43	CSTF2	GQGTLQHSPVGPAGPASIER	S8	0.67
44	CTNNA1	TSVQTEDDQLIAGQSAR	S2	**1.73**
45	CTNNB1	TSMoGGTQQQFVEGVR	T1	**1.42**
46	CTNND2	NSSLSPGLNTSNGDGSETETTSAILASVK	S5	0.53
47	D430041D05RIK	NVTASDEEEGAGLFDSAGK	S5	0.39
48	DBN1	LSSPVLHR	S3	0.03
49	DMTN	QPLTSPGSVSSSR	S5	0.65
50	DMXL2	SSDDIDYR	S2	0.45
51	DNAJC6	TATSASASPTLR	S8	0.33
52	DOPEY2	AALLAAFQPESPR	S11	0.08
53	DPYSL2	GLYDGPVCEVSVTPKTVTPASSAK	T13; T16; T18	0.48
54	DST	SGSASPAPGDTLPWNLPK	S3; S5	0.51
55	DYNC1I1	ADSEEEGAVELAA	S3	0.37
56	EIF4B	YAALSVDGEDEDEGDDCTE	T18	0.24
57	EIF4G2	TQTPPLGQTPQLGLK	T3	0.16
58	FAM63A	VETAEVCSRPQELPQSPR	S16	0.72
59	GAP43	EGDGSATTDAAPATSPK	S15	0.38
60	GPN1	GNASPVLDPSDLILTR	S4	0.51
61	GPR158	VSLPTANPDVSSGITQIK	S2	0.64
62	HCN1	LGTPPGGGAAGK	T3	0.38
63	HMGA1-RS1	EEEEGISQESSEEEQ	S10; S11	**7.85**
64	HN1	SNSSEASSGDFLDLK	S3	0.81
65	HSP90AB1	IEDVGSDEEDDSGKDKK	S6	0.29
66	HSPH1	IESPKLER	S3	0.04
67	HUWE1	AESPEEVACR	S3	0.57
68	IGF2R	AEALSSLHGDDQDSEDEVLTVPEVK	S14	0.55
69	KBTBD11	ASAAEGSEASPPSLR	S10; S13	0.34
70	KCNA2	IPSSPDLKK	S4	0.31
71	KCNMA1	RLEDEQPPTLSPK	T9; S11	0.06
72	KCNQ2	QNSEEASLPGEDIVEDNK	S3	0.55
73	KCTD12	SPSGGAAGPLLTPSQSLDGSR	S1	0.29
74	KIF1A	TPQPCSRPASPEPELLPELDSK	S6; S10	0.49
75	KPNA3	NVPQEESLEDSDVDADFK	S7	0.40
76	LMBRD1	SVIEGVDEDSDLSDDEPSAYSA	S10; S13	**11.30**

77	LMO7	EVIATEDVASLSSPTLPSSSLSHDR	S13	0.11
78	LMTK2	NAGFTSALLESPQR	S11	0.31
79	LSM12	TETPPPLASLNVSK	T3	0.30
80	MACF1	AFLAELEQNSPK	S10	0.33
81	MADD	SPTENVNTPVGK	S1	**1.41**
82	MAP6	AQSPLLPEPLK	S3	0.45
83	MARK1	SRPSSDLNNSTLQSPAHLK	S1; S4	0.59
84	MIOS	GFSQYGVSGSPTK	S10	0.77
85	MOBP	HQPAASPVVVR	S6	0.16
86	MPRIP	AEEQLPPLLSPPSPSTPHSR	S13	0.30
87	MYO5A	AISPTSATSSGR	S3	0.29
88	NCAM1	NPPEAATAPASPK	S11	0.24
89	NCAM2	DGSKEPIVEMoR	S3	0.22
90	NCOA5	LLRSSADSLPGELR	S5; S8	0.23
91	NDEL1	SAPSSPTLDCEK	S1; S4	0.54
92	NRBP2	EREDESEDESDILEESPCGR	S10	**1.61**
93	OBSCN	AGLASFR	S5	0.27
94	PAK1	DVATSPISPTENNTTPPDALTR	S8	0.22
95	PCDH17	ALSPLLQEVPSASSSPTK	T17	0.54
96	PKP4	SAVSPDLHITPIYEGR	S4	0.34
97	PLCL1	KLPSESDLLEGEVTDEDEEAEMoSR	T14	0.39
98	PLEC	NDESQLSPATR	S7	0.40
99	PPME1	QCEGITSPEGSK	T6	0.22
100	PRKCG	SPTSPVPVPVM	S1; S4	0.32
101	PRRT3	RGSQPDAELDGAGTSLLR	S3	0.69
102	PSIP1	NLAKPGVTSTSDSEDEDDQEGEK	S9; S11; S13	**6.81**
103	PSIP2	QSNASSDVEVEEK	S6	0.51
104	PSMA3	ESLKEEDESDDDNMo	S9	0.54
105	PSMD9	LASNSPVLPQAFAR	S5	0.6
106	PTK2B	NSLPQIPTLNLEAR	S2	0.57
107	PTPRN2	SEQPEEVLSSEEETAGVEHVR	S9; S10	0.39
108	PURB	RGGGSGGGDESEGEEVDED	S11	0.42
109	RAB3IP	TLVLSSSPTSPTQEPLAAAK	T9	0.42
110	RALBP1	TEGYAAFQEDSSGDEAESPSK	S11; S12	**1.67**
111	RAP1GAP2	SETSSNPSSPEICPNK	S5; S8; S9	0.190
112	RBM25	LGASNSPGQPNSVK	S6	0.39
113	RCSD1	AIVSPFHSPPSTPSSPGIR	S8	0.09
114	RGS8	SDSCSDFTAILPDKPNR	S3	0.24
115	RRAS2	FQEQECPPSPEPTR	S9	**1.31**
116	RTN1	SPPVAMoETASTGMoAAVPDALDHSPSSTLK	S23	0.11
117	SAMD14	SLDEDEPPPSPLAR	S1	0.45

118	SCG2	EHLGPGSSQEMER	S7	0.31
119	SEPT8	SDIAAAQQSGMSLSNSK	S16	0.20
120	SERINC1	LTLTSDESTLIEDGNGR	S5	0.33
121	SGIP1	DDYLETLSSPK	S9	0.31
122	SHANK2	QSNVEDSPEKTCSIPIPTIIVK	T11	In WT only
123	SHANK3	SLGEEPVGGLGSLLDPAK	S1	0.52
124	SIK3	GPSPLVTMoTPAVPAVTPVDEESSDGEPDQEAVQR	S22; S23	**5.03**
125	SLC1A3	DVEMGNSVIEENEMK	S7	0.37
126	SLC23A2	SSLAETLDSTGSLDPQR	S12	0.43
127	SLC24A2	SPTAGDKDGPTLPVGSSSAACSCTSEECVAESKPR	S1	0.57
128	SLC43A2	LCLSTVDLEVK	S4	0.27
129	SLC8A2	GISALLLNQGNGDKK	S3	0.31
130	SNAP91	KPGNNEGSGAPSPLSK	S8; S12	0.64
131	STK32C	GSSAAAPPSSPPPGR	S10	0.38
132	STMN3	SPSDLSPESPVLSSPPK	S9; S13	0.18
133	STRN4	ASPGPGGLSGGESLLVK	S2	0.37
134	STXBP1	LNKTDEEISS	S10	0.20
135	SYAP1	DDNLPLTEAVRPKTPPVVIK	T14	0.29
136	SYN1	LPSPTAAPQQSASQATPVTQGQGR	S3	0.74
137	SYT1	DDDAETGLTDGEEKEEPKEEEK	T9	**1.44**
138	THRAP3	IDISPSTFR	S4	0.61
139	THUMPD1	FIDKDQQPSGSEGEDDDAEAALK	S9; S11	**1.25**
140	TMCC1	ALGVISNFQSSPK	S11	0.76
141	TNKS1BP1	SSGSLSPGLETEDPLEAR	S6	0.82
142	TOP2B	IVETINSDSDSEFGIPK	S9	0.55
143	TTC7B	VEQALSEVASSLQSSAPK	S10; S11	0.67
144	UBXN6	GKSPQLALR	S3	0.01
145	USP20	AVPIAVADEGESESEDDDLKPR	S12; S14	0.28
146	VDAC2	LTFDTTFSPNTGK	S8	0.29
